# Dual Shield: Bifurcated Coating Analysis of Multilayered WO_3_/BiVO_4_/TiO_2_/NiOOH Photoanodes for Sustainable Solar-to-Hydrogen Generation from Challenging Waters

**DOI:** 10.1021/acssuschemeng.3c06528

**Published:** 2024-02-12

**Authors:** Logu Thirumalaisamy, Zhengfei Wei, Katherine Rebecca Davies, Michael G. Allan, James McGettrick, Trystan Watson, Moritz F. Kuehnel, Sudhagar Pitchaimuthu

**Affiliations:** † SPECIFIC, Materials Research Centre, Faculty of Science and Engineering, Swansea University (Bay Campus), Swansea SA1 8EN, U.K.; ‡ Department of Physics, G T N Arts College, Dindigul, Tamil Nadu 624005, India; § Department of Chemistry, Swansea University, Singleton Park, Swansea SA2 8PP, U.K.; ∥ Fraunhofer Institute for Microstructure of Materials and Systems IMWS, Walter-Hülse-Strasse 1, Halle 06120, Germany; ⊥ Research Centre for Carbon Solutions (RCCS), Institute of Mechanical, Processing and Energy Engineering, School of Engineering and Physical Sciences, Heriot-Watt University, Edinburgh EH144AS, U.K.

**Keywords:** solar energy, hydrogen, WO_3_/BiVO_4_, photoelectrochemical, TiO_2_, NiOOH, passivation, co-catalysts, spray coating, sputtering, mine, water pollutants, sustainability

## Abstract

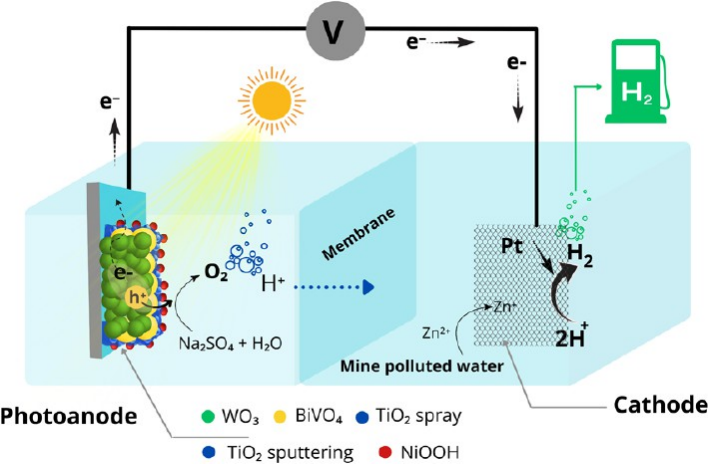

The heterostructure WO_3_/BiVO_4_-based photoanodes have garnered significant interest for photoelectrochemical (PEC) solar-driven water splitting to produce hydrogen. However, challenges such as inadequate charge separation and photocorrosion significantly hinder their performance, limiting overall solar-to-hydrogen conversion efficiency. The incorporation of cocatalysts has shown promise in improving charge separation at the photoanode, yet mitigating photocorrosion remains a formidable challenge. Amorphous metal oxide-based passivation layers offer a potential solution to safeguard semiconductor catalysts. We examine the structural, surface morphological, and optical properties of two-step-integrated sputter and spray-coated TiO_2_ thin films and their integration onto WO_3_/BiVO_4_, both with and without NiOOH cocatalyst deposition. The *J*–*V* experiments reveal that the NiOOH cocatalyst enhances the photocurrent density of the WO_3_/BiVO_4_ photoanode in water splitting reactions from 2.81 to 3.87 mA/cm^2^. However, during prolonged operation, the photocurrent density degrades by 52%. In contrast, integrated sputter and spray-coated TiO_2_ passivation layer-coated WO_3_/BiVO_4_/NiOOH samples demonstrate a ∼88% enhancement in photocurrent density (5.3 mA/cm^2^) with minimal degradation, emphasizing the importance of a strategic coating protocol to sustain photocurrent generation. We further explore the feasibility of using natural mine wastewater as an electrolyte feedstock in PEC generation. Two-compartment PEC cells, utilizing both fresh water and metal mine wastewater feedstocks exhibit 66.6 and 74.2 μmol/h cm^2^ hydrogen generation, respectively. Intriguingly, the recovery of zinc (Zn^2+^) heavy metals on the cathode surface in the mine wastewater electrolyte is confirmed through surface morphology and elemental analysis. This work underscores the significance of passivation layer and cocatalyst coating methodologies in a sequential order to enhance charge separation and protect the photoanode from photocorrosion, contributing to sustainable hydrogen generation. Additionally, it suggests the potential of utilizing wastewater in electrolyzers as an alternative to freshwater resources.

## Abstract 2

Concurrent recovery of heavy metals from mine-polluted water and green hydrogen generation at cathode through solar-driven photoelectrochemical process.

## 1 Introduction

In recent decades, humanity has grappled with a pressing energy predicament, necessitating the pursuit of environmentally sustainable energy alternatives. Solar energy, owing to its intrinsic attributes of decentralization and inexhaustibility, presents a compelling substitute for conventional fossil fuels. Nonetheless, the full integration of solar energy into the global energy infrastructure requires the attainment of several pivotal objectives. Within this context, the prospect of harnessing sunlight to synthesize fuel emerges as a promising strategy for meeting the energy demands of both industrial and residential sectors while concurrently mitigating the emissions of greenhouse gases. Photoelectrochemical (PEC) technology, employing photoactive semiconductors in conjunction with appropriate electrolytes, such as water or carbon dioxide (CO_2_), has garnered considerable attention as a potent means of converting solar radiation into hydrogen fuel.^1^ Analogous to the process of natural photosynthesis, the phenomenon of PEC water oxidation (H_2_O + 2h^+^ → 1/2O_2_ + 2H^+^), when coupled with light and semiconductors, offers innovative avenues for the cost-effective production of clean hydrogen fuel (2H^+^ + 2e^–^ → H_2_) utilizing water as the source material.^2^ The charge carriers (electrons and holes, represented as e^–^ and h^+^) generated when semiconductors that are exposed to light play a pivotal role in initiating the water-splitting reaction. This process leads to the production of oxygen and hydrogen fuel.^3^ To harness the solar energy for water-splitting reactions, the semiconductors with visible light bandgap energy is appropriate which can absorb a significant portion of the sunlight’s photons, making them effective in converting solar energy into chemical energy through water splitting.^4^

Earth-abundant metal oxides, including ZnO,^5^ TiO_2_,^6^ WO_3_,^7^ Fe_2_O_3_,^8^ and BiVO_4_,^9^ are currently under investigation as prime candidates within the semiconductor photoanode family. BiVO_4_, in particular, exhibits promising characteristics, with the potential to achieve a photocurrent density (PCD) of up to 7.5 mA cm^2^ at 1.23 V reversible hydrogen electrode (RHE).^10^ This capability stems from its effective defect tolerance and moderate charge transport properties, particularly under simulated AM 1.5G illumination.^10^ WO_3_ and BiVO_4_ are notably attractive as photoanode materials due to their defect tolerance, cost-effectiveness, and desirable narrow band gap energy.^11^ However, these materials alone do not yield sufficiently high solar-hydrogen conversion efficiency for practical applications. The heterostructure combination of WO_3_/BiVO_4_ photoanode demonstrates effective charge separation compared to individual photoanodes of WO_3_ and BiVO_4_.^12,13^ This is because BiVO_4_ exhibits time-scale-dependent charge transfer characteristics,^14^ necessitating a partner material to separate the photoelectrons from the conduction band of BiVO_4_ toward the charge collector. Additionally, BiVO_4_ faces challenges related to defect formation led photocorrosion during prolonged PEC reaction processes, frequently resulting in V^5+^ leaching from the BiVO_4_ lattice.^15,16^ The establishment of a type-II WO_3_/BiVO_4_ heterojunction stands out as a exemplary solution for alleviating the inadequate electrical properties of BiVO_4_ and the limited potential-harvesting capacity of WO_3_.^17^ To further enhance the PEC activity of WO_3_/BiVO_4_, several strategies have been investigated, including cocatalysts deposition,^18,19^ morphology modification,^20^ doping,^21^ and the incorporation of interfacial layers.^22^ Achieving potentially high PEC performance with long-term durability necessitates the post alteration of a WO_3_/BiVO_4_ heterojunction with a surface protective agent.^23^

Among these options, the construction of a passivation layer such as NiCo_2_O_*x*_,^24^ Bi_2_S_3_,^25^ CoP,^26^ BiFeO_3_,^27^ ZnO,^28^ TiO_2_^29^ on top of a WO_3_/BiVO_4_ heterojunction has demonstrated effective performance in enhancing stability. In a study conducted by Wei et al.,^24^ a robust catalyst known as NiCo_2_O_*x*_ was applied to the surface of WO_3_/BiVO_4_ photoanodes. This catalyst exhibited a favorable photocurrent of 3.85 mA cm^2^ at 1.23 V vs. RHE and maintained excellent stability over a 3 h period. Another approach involved the utilization of a heterostructure nanoarray consisting of Bi_2_S_3_/BiVO_4_/WO_3_, which led to improved PEC hydrogen evolution.^25^ The CoP/BiVO_4_/WO_3_ electrode displayed a highly stable PCD for at least 5 h.^26^ Additionally, BiFeO_3_ was introduced into the WO_3_/BiVO_4_ system resulting in high PEC performance and stability.^27^ To achieve an excellent PEC performance, sustained photoactivity, and effective passivation of sublayers, a ZnO layer introduced onto the WO_3_/BiVO_4_ type-II heterojunction.^28^ However, coating TiO_2_ layer played a crucial role in protecting BiVO_4_ against photocorrosion and degradation.^30^ Surface post modification with a nanometer-thick layer of single-crystalline TiO_2_ yielded stable PCD up to 1.04 mA cm^2^ at 1.23 V and long-term photostability (24 h).^23^ Despite the significant advancements achieved in previous investigations, various overlayer materials were used, with limited success in achieving sufficient stability. In comparison, passivation layers based on amorphous metal oxides provide a promising solution for protecting semiconductor catalysts, with TiO_2_ demonstrating particularly encouraging results. A diverse array of coating methods is employed for depositing TiO_2_ passivation layers.

Among the various approaches for synthesizing low-density thin films, spray pyrolysis stands out as the most versatile technique for depositing TiO_2_ thin films.^31^ This method offers the advantage of producing highly crystalline and well-structured films.^32^ Several parameters in this technique influence the characteristics of the deposited film, including the nozzle-to-substrate distance, droplet diameter, precursor composition/concentration, substrate temperature, flow rate, deposition time, and carrier gas. Adjusting these experimental parameters allows for flexibility in tailoring the properties of the film, making spray deposition a versatile choice. Moreover, the affordability of fabricating films using spray pyrolysis has contributed to its popularity. This technique also enables precise control over thin film morphology and particle size on a nanometer scale.^33,34^

However, one of the main challenges in spray coating of metal oxides is achieving compact and pore-free thin films, especially when compared to vacuum-based physical coating methods. Vacuum-based techniques such as chemical vapor deposition (CVD), atomic layer deposition, and direct current (DC) or radio frequency (RF) magnetron sputtering are promising for producing robust and conformal TiO_2_ coatings. Sputtering techniques, in particular, have garnered attention due to their stability, reproducibility, ease of instrumentation handling, and control over a range of substrates.^35^ Nevertheless, it is worth noting that materials cost can be higher with vacuum-based coating techniques compared to spray processing films. Therefore, exploring an integrated approach that combines the merits of both techniques could lead to cost-effective passivation layers for photoanodes.

To enhance charge separation and transfer, various practices have been employed, including the use of passivation layers and cocatalysts. Recent reports suggest that the PEC activity of the WO_3_/BiVO_4_ heterojunction can be further improved by incorporating oxygen evolution catalysts, which enhance charge transfer kinetics at the electrode/electrolyte interface. Oxygen evolution catalysts such as NiOOH, FeOOH, and CoO_*x*_ have been employed to enhance the PEC performance of the WO_3_/BiVO_4_ heterojunction photoanode.^36−38^ Additionally, the oxygen vacancies within these cocatalysts for oxygen evolution may serve as external driving forces for hole trapping and facilitate highly oxidizing hole migration, thereby reducing energy losses at the intrinsic potential barrier at the photoanode/electrolyte interface.

In this work, we propose a novel approach to enhance the PEC performance of a WO_3_/BiVO_4_ heterojunction for solar-driven water splitting, addressing challenges such as inadequate electrical properties of BiVO_4_, limited potential-harvesting capacity of WO_3_, and the need for stable passivation layers. The key novelty lies in the synergistic combination of two coating techniques, namely spray and sputter-coated TiO_2_ thin films, as a protective passivation layer for the WO_3_/BiVO_4_ photoanode. The study also explores the impact of NiOOH cocatalyst deposition on the photoanode. The integration of both techniques aims to leverage the advantages of each, offering a cost-effective and precise solution for achieving compact and pore-free thin films. This work employs a “bifurcated coating analysis” approach, first examining the influence of the passivation layer on PEC performance under varying processing parameters and then investigating the impact of NiOOH cocatalysts, providing a comprehensive understanding of the contributions of each coating component to the WO_3_/BiVO_4_ system. This innovative strategy addresses existing challenges in stabilizing semiconductor catalysts, contributing to the advancement of solar-driven hydrogen production technologies. Furthermore, this work introduces a noteworthy contribution by demonstrating the feasibility of PEC hydrogen generation using real-time mine water pollutants instead of conventional freshwater-based electrolytes. This additional aspect not only broadens the application scope of the proposed technology but also addresses environmental concerns related to water usage in energy conversion processes.

## 2 Experimental Section

### 2.1 Photoanode Preparation

A photoanode with a surface area of 1 cm^2^ was fabricated with the configuration FTO/WO_3_/BiVO_4_/TiO_2_/5 nm sputtered TiO_2_. For this, initially, FTO was cleaned with a soap solution and double-distilled water and ultrasonicated for 10 min in acetone and isopropyl alcohol (IPA) separately. Lastly, UV–ozone treatment was carried out to eliminate organic impurities.

#### 2.1.1 Preparation of WO_3_ Slurry and Nanocrystalline Porous Films

The multistage synthesis procedure for preparing the mesoporous WO_3_ slurry used in photoanode fabrication is as follows: an ethyl cellulose solution was meticulously prepared by stirring 1.5 g of 30–60 mPa s ethyl cellulose and 1.5 g of 5–15 mPa s ethyl cellulose (Sigma-Aldrich) in 27 g of ethanol overnight. Tungsten(IV) oxide powder (5 g) was ground with 1 mL of acetic acid for 5 min, followed by adding 1 mL of deionized water and grinding for 1 min, repeating this step six times. Subsequently, 1 mL of ethanol was added and ground for 15 repetitions. Further, 2.5 mL of ethanol was added and ground for 6 repetitions. The resulting slurry was then diluted in 100 mL of ethanol for sonication, avoiding nanoparticle aggregation through ultrasonication with an “outgas” pulsating function for 30 s, followed by 1 min of magnetic stirring. To this mixture, 20 g of α-terpineol (Sigma-Aldrich) was added and stirred for 1 min, repeating the “outgas” pulsating process. Finally, 20 g of preprepared ethyl cellulose was added and stirred for 1 min, followed by repeating the “outgas” pulsating process. The ethanol was evaporated using a rotary evaporator until the desired viscosity was achieved. The resulting WO_3_ paste was collected for deposition onto a fluorine-doped tin oxide (FTO) substrate using the doctor blade technique, and the bottom WO_3_ mesoporous layer with a thickness of approximately 5.5 μm was coated. The coated substrate was annealed at 450 °C for 3 h to complete the process.

#### 2.1.2 Preparation of WO_3_/BiVO_4_ Heterojunction

A BiVO_4_ layer was applied onto a precoated WO_3_/FTO substrate by spin coating technique. In a standard synthesis procedure, a mixture was prepared by combining 0.1462 g of ammonium metavanadate, 0.6061 g of bismuth nitrate pentahydrate, 0.4803 g of citric acid, 0.825 g of nitric acid, and 2.9 mL of deionized water. This mixture was then sonicated for 30 min to ensure the complete dissolution of the precursor materials. Subsequently, the BiVO_4_ layer was deposited onto the WO_3_ substrate by spin coating the solution at 3000 rpm for 40 s, followed by annealing at 450 °C for 1 h, with a ramping period of 3 h.

#### 2.1.3 Preparation of WO_3_/BiVO_4_/Sprayed TiO_2_

Following this step, a spray technique was employed to apply a ∼130 nm thick, low-density TiO_2_ layer onto the FTO/WO_3_/BiVO_4_ structure. The precursor solution, consisting of TiAcAc dissolved in isopropanol (Sigma-Aldrich), was mixed with a ratio of 1:9, and this solution was then sprayed onto a glass substrate at the microscopic level. The growth of the TiO_2_ low-density thin films was achieved using the spray pyrolysis technique, with deposition conducted at various substrate temperatures (150, 200, and 250 °C). Throughout each deposition, the nozzle-to-substrate distance was maintained at 15 cm. Parameters such as the nozzle-substrate distance, carrier gas pressure, spray time, and spray rate were carefully optimized to ensure the production of high-quality TiO_2_ thin films. Subsequently, the deposited films underwent annealing at 450 °C for 3 h.

#### 2.1.4 Preparation of WO_3_/BiVO_4_/Sprayed TiO_2_/Sputtered TiO_2_

To enhance the film integrity of TiO_2_ spray coated layer, a ∼5 nm very thin and high dense TiO_2_ layer was prepared on top of the WO_3_/BiVO_4_/sprayed TiO_2_ by RF magnetron sputtering at room temperature and annealed at 450 °C for an hour. Nanoporous TiO_2_ was RF sputtered at room temperature with a power density of 2.26 W cm^–2^ using a high vacuum Moorfield Minilab 60 sputtering system. Sputtered TiO_2_ were deposited at 5, 10, 15, and 31 nm respectively, followed by a post annealing process at 450 °C in air. For comparison purposes, we prepared WO_3_/BiVO_4_ photoanodes with integrated TiO_2_ films, achieved through a combination of spray and sputter deposition techniques.

#### 2.1.5 Electrochemical Synthesis of NiOOH onto Photoanode

To prepare Ni(OOH) catalysts, electrodeposition was performed on the WO_3_/BiVO_4_/sprayed TiO_2_/sputtered TiO_2_ photoanode. For NiOOH electrodeposition, a solution of 0.025 M nickel nitrate hexahydrate (Alfa Aesar, 99.9985%) was dissolved in Milli-Q water and used as the electrolyte. In the electrochemical deposition process, the prefabricated photoanode WO_3_/BiVO_4_/sprayed TiO_2_/sputtered TiO_2_ obtained from Section 2.1.4 served as the working electrode. A reference electrode of Ag/AgCl and a counter electrode made of platinum were employed. The reaction was carried out under a constant potential of 0.8 V, applied for a duration of 15 min. After the electrodeposition, the newly deposited films were rinsed with Milli-Q water to eliminate any residual electrolyte, followed by drying using nitrogen gas. Periodic cleaning of the platinum counter electrode was performed using 30% nitric acid.

To establish electrical contact from the photoanode to the collector, an ohmic contact was established for the WO_3_/BiVO_4_/sprayed TiO_2_/sputtered TiO_2_ photoelectrode with an active area of 1 cm^2^. This was accomplished by soldering a copper (Cu) wire onto the FTO surface of the sample using ultrasonic soldering and securing it in place with adhesive epoxy resin.

### 2.2 Characterization

The changes in crystallographic behavior result from variations in substrate temperature affecting the spatial lattice of TiO_2_ deposited through both spraying and sputtering techniques. These changes were explored using (XRD) X-ray diffraction, employing a Bruker D8 Discover X-ray diffractometer with a copper source (40 kV, 40 mA) and a 1D detector in Bragg–Brentano geometry. For an investigation into surface morphologies, field emission scanning electron microscopy (FESEM) was employed. Specifically, a JEOL 7800F FEGSEM equipped with an Oxford Instrument X-MaxN energy dispersion spectra (EDS) detector featuring a 50 mm^2^ window was utilized. The chemical composition of the thin films was analyzed through X-ray photoelectron spectroscopy, employing the Kratos Axis Supra instrument with a monochromatic Al K X-ray source operating at 225 W (15 mA emission current). This analysis was conducted to identify the presence of elements and their oxidation states. To determine the thickness of the produced thin films, an Ambios XP2 surface profiler was employed. The optical absorption characteristics of the photoactive layers WO_3_, BiVO_4_, with passivation layers were assessed using a PerkinElmer Lambda 365 spectrometer.

### 2.3 Photoelectrochemical Measurements

All PEC measurements were conducted using an Autolab PGSTAT 302N electrochemical station, and the NOVA software was employed to control and operate these measurements. For linear cyclic voltammetry (LSV) and chronoamperometry experiments, a single-electrode chemical cell setup made of glass was utilized. The working electrode was the WO_3_/BiVO_4_ photoanode, modified with TiO_2_ passivation layers and NiOOH cocatalysts, with an active area of 1 cm^2^. The counterelectrode was a platinum (Pt) mesh, and an Ag/AgCl electrode served as the reference electrode. A 0.5 M aqueous Na_2_SO_4_ electrolyte was used for all PEC studies. PEC experiments were conducted using a class AAA solar simulator (350–1100 nm) equipped with a built-in AM 1.5G filter (ASAHI SPECTRA, Japan). One sun illumination was verified using the ASHAI SPECTRA 1 sun checker, which employed both a silicon photodiode and an InGaAs PIN diode. It is important to note that all PEC experiments were performed with front-side light illumination on the photoanode.

### 2.4 Hydrogen Quantification

Measurements of the evolution of hydrogen gas were made in a two-compartment electrochemical cell setup made of glass under one sun’s light using a solar light simulator (Thermo Oriel 92194-1000) fitted with a Newport AM 1.5G filter. The prepared WO_3_/BiVO_4_/sprayed TiO_2_/sputtered TiO_2_ and WO_3_/BiVO_4_/sprayed TiO_2_/sputtered TiO_2_/NiOOH electrodes were the photoanodes employed in the anode compartment. The reference electrode was an Ag/AgCl electrode, whereas the counterelectrode was a platinum (Pt) mesh. We employed 0.5 M aqueous Na_2_SO_4_ as the electrolyte. A Nafion membrane divided the anode and cathode compartments, and a rubber stopper effectively sealed the cathode compartment. Nitrogen gas (N_2_) was continuously purged from the sample headspace at a rate of 10 mL min^–1^. Gas chromatography (GC) (Shimadzu Nexis 2030) was used to track the evolution of hydrogen (H_2_), and an autosampler was set up to continuously inject 2 mL of the headspace stream into the system. Prior to injection, the gas samples were passed through a 2 mL sample loop (Restek). The measured H_2_ content in the purge gas and the purge gas flow rate were used to compute the hydrogen evolution rates. A comprehensive protocol for quantifying hydrogen is outlined in our previous reports.^39,40^

## 3 Result and Discussion

### 3.1 Crystallite Structure, Surface Morphology Analysis

**Figure 1:**
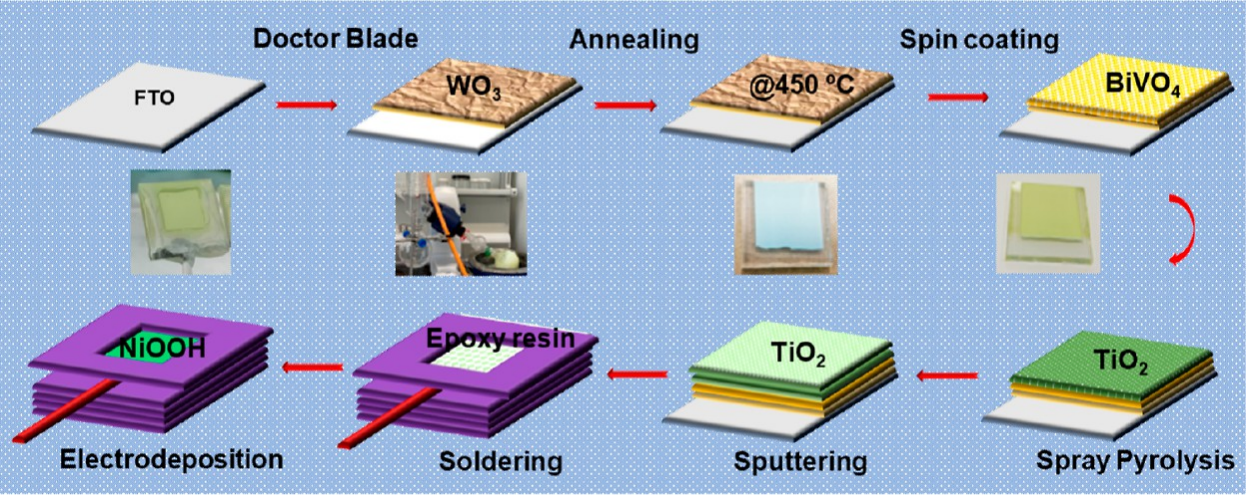
Schematic depiction of WO_3_/BiVO_4_ photoanode fabrication with post-deposition of TiO_2_ and NiOOH.

**Figure 2:**
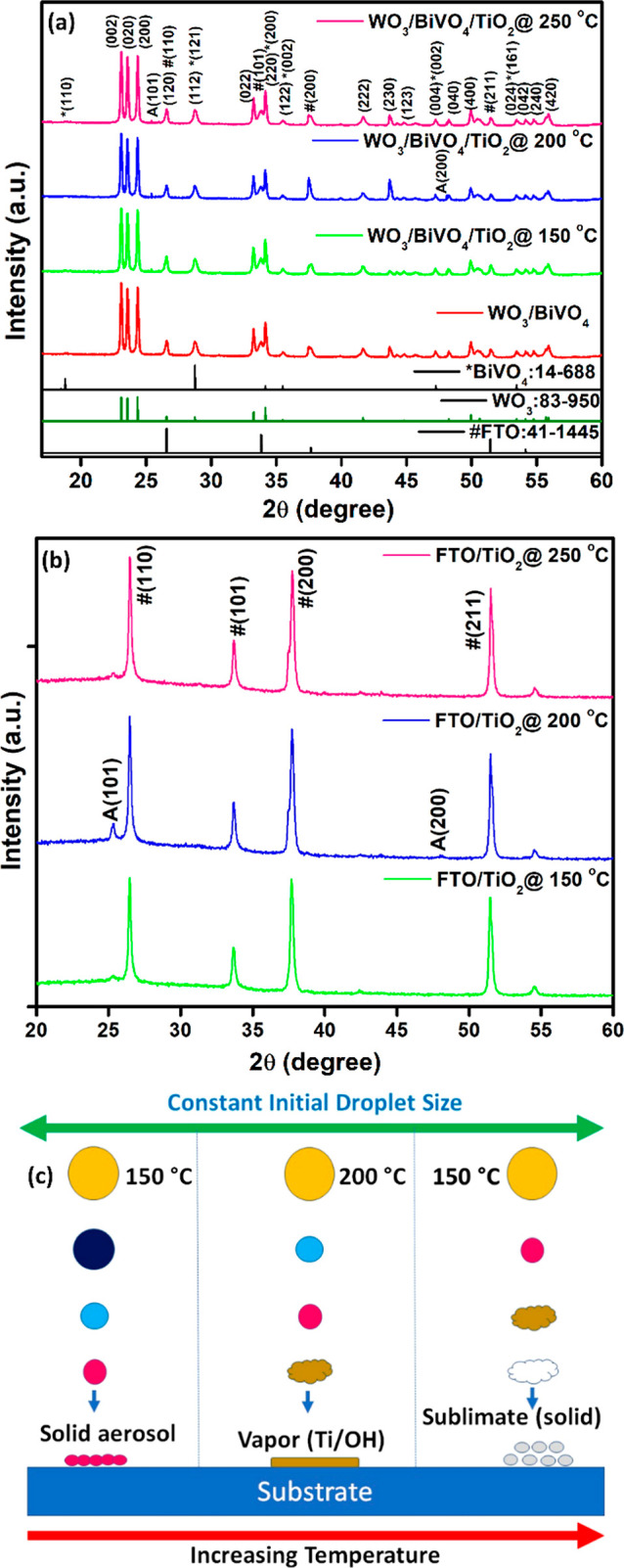
(a) XRD patterns for the WO_3_/BiVO_4_ and WO_3_/BiVO_4_/TiO_2_ films (deposited at different substrate temperature 150, 200 and 250 °C). (b) XRD pattern of spray coated TiO_2_ on bare FTO substrate. (c) Aerosol decomposition mechanism for different substrate temperature.

The XRD patterns for the WO_3_/BiVO_4_ and WO_3_/BiVO_4_/TiO_2_ (deposited at 150, 200 and 250 °C) electrodes were recorded and are shown in Figure 2a1–a4. The XRD peaks were indexed using Powder X software and revealed that no identification of binary or impurity phases in all prepared electrodes. Figure 1a1 illustrates the diffraction peaks at 23.1, 23.6, 24.4, 26.6, 28.9, 33.3, 34.2, 35.7, 41.9,43.7, 44.7, 47.3, 48.3, 49.9, 53.5, 54.2, 54.8, and 55.9°; these peaks assigned to the (002), (020), (200), (120), (112), (022), (220), (122), (222), (230), (123), (004), (040), (400), (024), (042) (240), and (420) planes, correspondingly, signifying monoclinic WO_3_ phase and well matched with the JCPDS card # 83-950.

The WO_3_/BiVO_4_ heterojunction showed continuance of the WO_3_ phase and Bragg diffractions at 18.7, 28.9, 34.5, 35.2, 47.3, and 53.4°; these peaks correspond to the (110), (121), (200), (002), (042), and (161) planes, respectively, portraying the generation of a monoclinic BiVO_4_ form. The diffraction patterns of BiVO_4_ are consistent with the JCPDS card 14-688. As illustrated in Figure 2a the spray coated TiO_2_ at different substrate temperature show a weak crystallite peak at 25.4 and 48.1°, corresponds to (101), (200) planes confirming the generation of anatase TiO_2_ phase (JCPDS # 21-1272). However, their crystallite peak is very weak compared with WO_3_ and BiVO_4_.

To gain a deeper insight into the crystalline characteristics of TiO_2_, we prepared spray-coated TiO_2_ films at different substrate temperatures. The X-ray diffraction (XRD) pattern of the spray-coated TiO_2_ on the bare FTO substrate at varying substrate temperatures (Figure 2b) revealed the presence of weak peaks at 25.4 and 48.1°, indicating the existence of (101) and (200) planes of anatase TiO_2_. The XRD patterns further indicated that the deposited TiO_2_ films possessed a polycrystalline nature. Notably, among the various films, the one deposited at 200 °C exhibited an enhanced level of crystallinity. Conversely, a reduction in the intensity of peaks related to crystalline planes (101) and (200) was observed for films deposited at 150 and 250 °C. However, the film deposited at 200 °C displayed relatively more intense and well-defined diffraction peaks compared to those obtained under the other two substrate temperature conditions (150 and 250 °C). The XRD analysis of the TiO_2_ coating, which is 5 nm thick and applied onto the FTO substrate, does not reveal any crystalline peaks, indicating its amorphous nature (see Figure S1).

We can explain this behavior through the Viguié and Spitz spray mechanism^41,42^ (Figure 2c). At 150 °C, when large droplets approach the substrate, these aerosol particles splash onto the surface, followed by the precipitation of an amorphous salt (Ti[OH]_4_), resulting in a film with low crystallinity. Conversely, in the case of 250 °C, either very small droplets are initially formed or the entrainment process leads to more extensive evaporation. This causes the entrained aerosol particles to precipitate as amorphous salt and sublime or oxidize well before reaching the substrate, resulting in poor adherence to the substrate and, consequently, a film with low crystallinity. However, at 200 °C, when small droplets are initially formed or the entrainment process causes extensive evaporation, these entrained aerosol particles precipitate as amorphous salt and then sublime immediately before reaching the substrate. Vapor transport to the substrate surface results in subsequent decomposition/oxidation, leading to the formation of a high-quality crystalline film. Based on these findings, it is evident that a substrate temperature of 200 °C is the most suitable to achieve a low thickness and high-quality crystalline TiO_2_ film.

**Figure 3:**
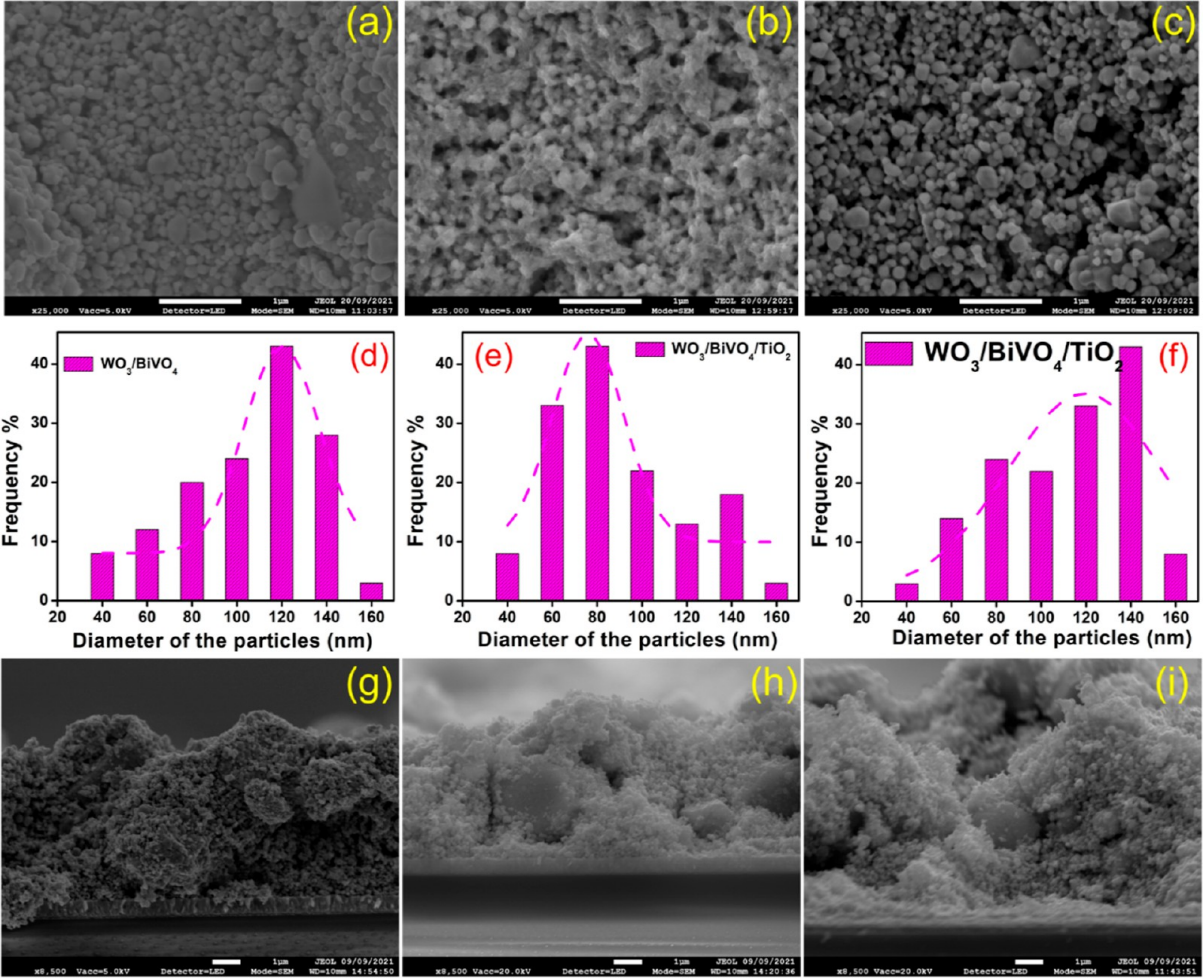
FESEM images of (a) WO_3_/BiVO_4_, (b) WO_3_/BiVO_4_/TiO_2_@200 °C, and (c) WO_3_/BiVO_4_/TiO_2_@250 °C film. Corresponding particle size analysis results are presented in (d–f), respectively. The cross section FESEM images of (g) WO_3_/BiVO_4_, (h) WO_3_/BiVO_4_/TiO_2_@200 °C, and (i) WO_3_/BiVO_4_/TiO_2_@250 °C film.

The SEM and cross-sectional SEM images of WO_3_/BiVO_4_ and WO_3_/BiVO_4_/TiO_2_ films prepared at 200 and 250 °C are presented in Figure 3. In Figure 3a, it is evident that the WO_3_/BiVO_4_ particles exhibit a spherical shape, with particles interconnected (mesoporous structure). The average particle sizes, as estimated from Figure 3a–c, are summarized in Figure 3d–f. These results illustrate that the WO_3_/BiVO_4_ particles show variations in particle size due to the presence of the spray-coated TiO_2_. The cross-sectional SEM images (Figure 3g–i) offer additional insights into the interfaces between the film and the substrate. They reveal that WO_3_/BiVO_4_ adheres well to the FTO substrate, and the particles forming the coating exhibit a mesoporous structure. These films exhibit a thickness ranging from 4 to 6 μm. We have conducted precise thickness measurements of the photoanode for various coating configurations using a surface profilometer. The averaged values obtained from three independent measurements are presented in Figure S2.

Figure 3a–c revealed that the significant pores exhibit between neighboring WO_3_ particles. These mesopores originate during the thin film processing stage, particularly when removing ethyl cellulose binders from the WO_3_ nanoparticles, resulting in the formation of large mesopore channels. An illustrative example of this phenomenon can be found in our recent work on WO_3_/BiVO_4_.^39^ Additionally, the application of a thin BiVO_4_ layer on WO_3_ through spin coating covers the WO_3_ surface, creating interconnected channels within the WO_3_/BiVO_4_ network. The presence of these pore channels is crucial as they facilitate the easy percolation of the electrolyte through the photoanode. This enhanced mass transport within the mesoporous channels plays a pivotal role in promoting higher charge carrier separation, as demonstrated in many studies.^27,43^ Therefore, the mesoporous structure of our WO_3_/BiVO_4_ particles is not merely an incidental characteristic but a deliberate design element that significantly contributes to improved PEC performance.

Additionally, energy dispersive X-ray spectroscopy (EDS) elemental maps (refer to the Supporting Information Figure S3) were obtained to assess the uniform distribution of W, Bi, V, O, and Ti elements across the entire structure (WO_3_/BiVO_4_/TiO_2_).^44^

### 3.2 Optical Properties

**Figure 4:**
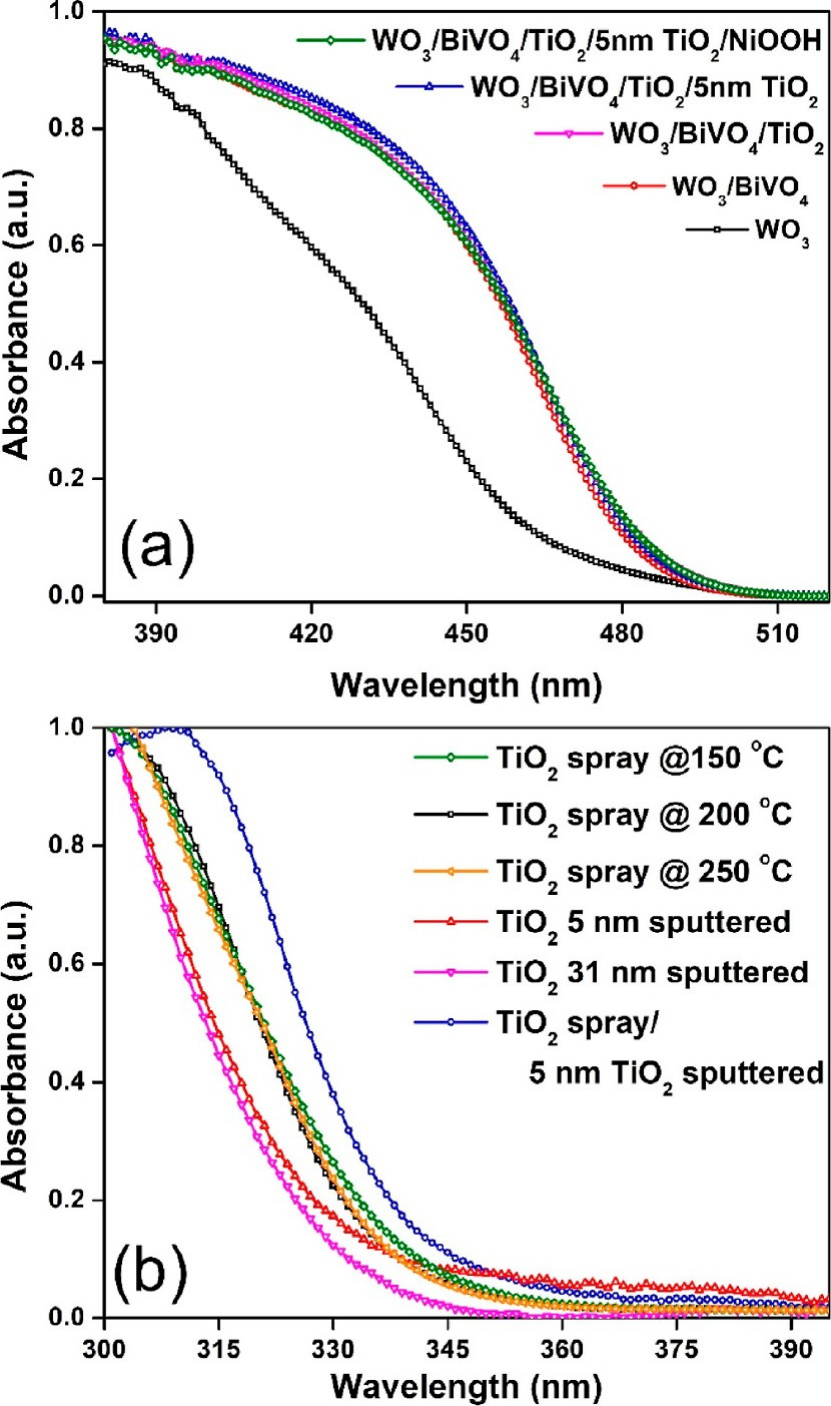
Absorption spectra of (a) WO_3_ and WO_3_/BiVO_4_ films with and without TiO_2_ and NiOOH coatings, and (b) the absorption spectra of TiO_2_ thin films synthesized through spray coating at various substrate temperatures and sputtering coating at different thicknesses.

Ultraviolet–visible (UV–vis) absorption spectroscopy was employed to gain insights into the photon absorption properties of the prepared electrodes. As illustrated in Figure 4a, bare WO_3_ films exhibited strong photon absorption within the range of 380–470 nm. In contrast, the incorporation of BiVO_4_ on WO_3_, it extended the absorption wavelength to 380–500 nm, indicating an enhanced photon absorption capability. This heightened light harvesting, achieved by incorporating the BiVO_4_ layer onto the primary WO_3_ photoabsorber, has the potential to increase photon reception at two distinct wavelengths. This, in turn, can accelerate the catalytic reaction rate for water oxidation reactions. Specifically, an increased concentration of light photons reaching the catalytically active sites translates to higher rates of water oxidation, leading to the generation of oxygen gas and byproducts (protons). Consequently, this process indirectly amplifies hydrogen evolution at the cathode. This trend remained consistent even with the addition of TiO_2_ coatings, whether through the spray or sputtering methods, as well as NiOOH coatings. To further investigate the impact of TiO_2_ synthesis methods on its optical behavior, we recorded the absorbance spectra of spray-coated TiO_2_ at various substrate temperatures and sputter-coated TiO_2_ films of different thicknesses, as shown in Figure 4b. Notably, the absorption edges of spray-coated TiO_2_ at different substrate temperatures were nearly identical. However, the absorption edges of sputter-coated TiO_2_ shifted toward shorter wavelengths with increasing film thicknesses (ranging from 5 to 31 nm), indicating crystal growth.^45^ An intriguing observation was that the absorption edge of two-step spray-coated and sputter-coated TiO_2_ thin films shifted to shorter wavelengths compared to single coatings applied using either the spray or sputtering technique, thereby enhancing the photon absorption capability. Possible reasons for this blueshift may include the increased volume of TiO_2_^31^ or the influence of impurities that could alter the valence of the Ti,^32^ affect the oxygen content,^33^ and introduce structural disorder.^34^

### 3.3 Photoelectrochemical Studies

**Figure 5:**
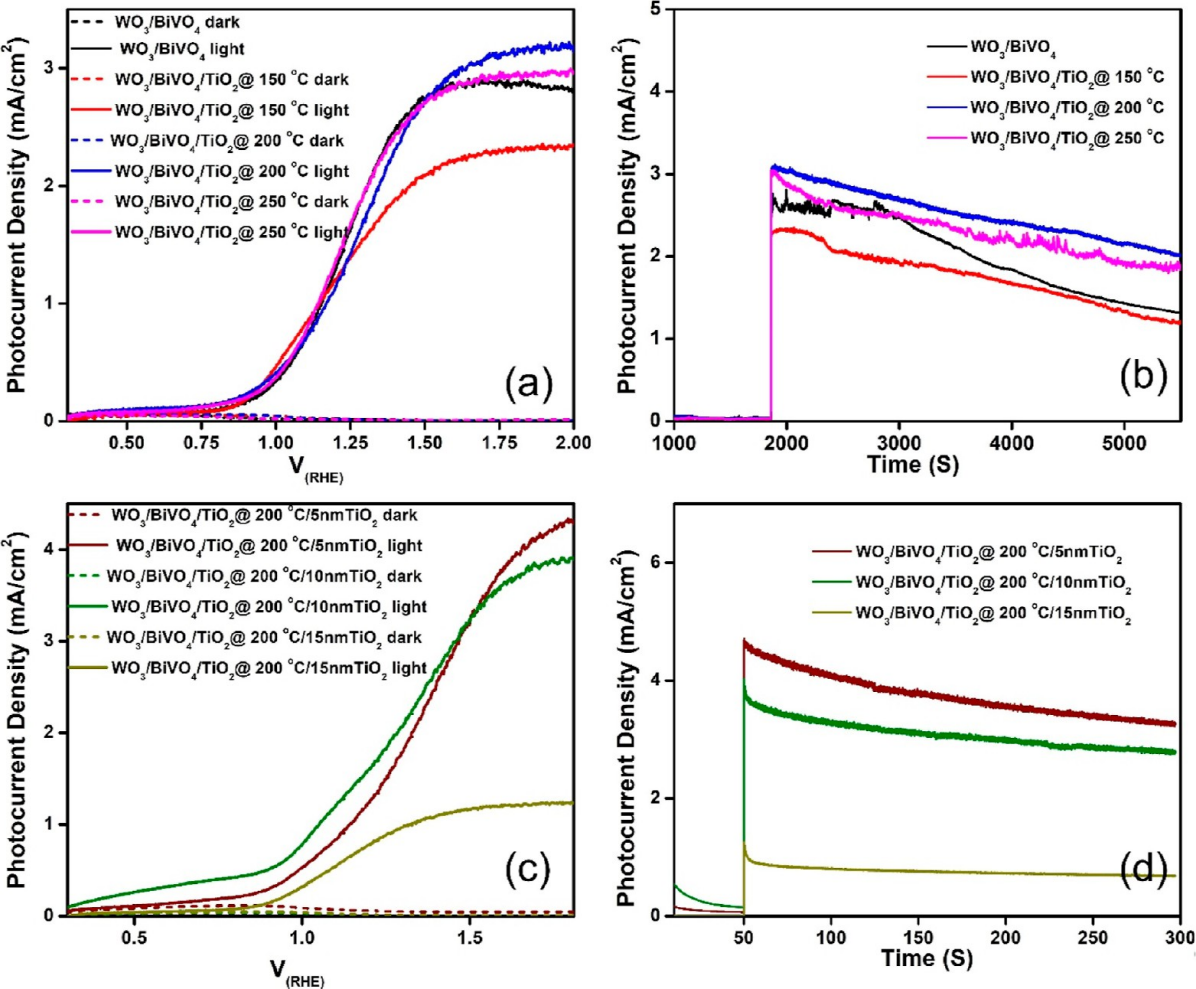
(a) *J*–*V* plot and (b) chronoamperometry of WO_3_/BiVO_4_, and WO_3_/BiVO_4_/TiO_2_ at different substrate temperature photoanodes. (c) *J*–*V* plot and (b) chronoamperometry of PEC cells with WO_3_/BiVO_4_/sprayed TiO_2_/sputtered TiO_2_ photoanode (for different thickness of TiO_2_ films). Note that the electrolyte is 0.5 M aqueous Na_2_SO_4_.

The performance evaluation of TiO_2_ passivation coatings, synthesized at varying spray processing temperatures, was conducted in photoelectrocatalytic water splitting reaction. In Figure 5a, we present the *J*–*V* plots for the WO_3_/BiVO_4_ photoanode, both with and without TiO_2_ passivation coatings, under dark and light irradiation. From the data shown in Figure 5a, it becomes evident that no current is generated under dark irradiation. In the case of light irradiation, the photoanode showed photocurrent generation significantly, ensuring the PEC effect. Briefly, under light irradiation, the photoholes generated at the valence band of the WO_3_/BiVO_4_ photoanode oxidize water into oxygen gas, commencing at a minimum onset potential of approximately 0.8 V RHE. Simultaneously, the photoelectrons generated from conduction band of the WO_3_/BiVO_4_ photoanode transport to the Pt cathode, facilitating the reduction of protons into hydrogen gas. The WO_3_/BiVO_4_ photoanode achieves a photocurrent generation 2.81 mA/cm^2^ performance at 2 V RHE. Furthermore, by depositing TiO_2_ thin films via spray coating at 200 °C, the PCD is enhanced by 14%, reaching approximately 3.19 mA/cm^2^ at 2 V RHE. However, photoanodes coated with TiO_2_ thin films, spray-coated at 250 °C, exhibit a reduced PCD of 2.3 mA/cm^2^. The reduction observed above the substrate temperature of 250 °C can be attributed to the thickness of the TiO_2_ passivation layer. A thicker film synthesized at 250 °C, may block the hole transport from BiVO_4_ layer to electrolyte.

As a result, the TiO_2_ passivation layer-coated WO_3_/BiVO_4_ photoanode demonstrates good stability for up to an hour, with a 32% reduction in current (Figure 5b). This performance surpasses that of TiO_2_ uncoated photoanodes, which experience a 53% reduction. It is worth appreciating that the TiO_2_ thin film layer serves as protection against the well-known issue of photocorrosion, wherein vanadium vacancies are observed leaving the BiVO_4_ lattice. However, the 32% reduction in photocurrent suggests the need to improve the coverage of TiO_2_ spray-coated films, as there may be uncovered TiO_2_ sites on the BiVO_4_ surface. The presence of uncovered TiO_2_ sites, including voids and pinholes, on the BiVO_4_ surface due to spray coating may potentially enable electron backflow from the WO_3_/BiVO_4_ anode to the electrolyte, resulting in electron leakage. A similar issue has been previously reported in spray-coated TiO_2_ thin films used as electron transport layers in perovskite solar cells^46−48^ and as blocking layers in dye/QDs-sensitized solar cells.^49−51,51^ The optimized WO_3_/BiVO_4_ photoanode, configured with a spray-coated TiO_2_ thin film at 200 °C, underwent further deposition with different thicknesses (5, 10, and 15 nm) of sputtered TiO_2_ thin films.

Figure 5c illustrates the *JV* plots of WO_3_/BiVO_4_/TiO_2_ (spray) photoanodes with varying thicknesses (5, 10, and 15 nm) of sputtered TiO_2_ thin films. The secondary coating of sputtered TiO_2_ films onto WO_3_/BiVO_4_/TiO_2_ (spray) significantly enhances photocurrent generation, increasing it from approximately 3.19 mA/cm^2^ to about 4.3 mA/cm^2^ (Figure 5c). This improvement is likely due to the enhanced coverage of the passivation layer. However, when the thickness is increased further from 5 to 10 nm, the PCD tends to decrease. The thickness of the sputtered TiO_2_ layer plays a crucial role in facilitating the hole transport from the valence band of WO_3_/BiVO_4_ to the electrolyte, enabling the tunneling effect. The tunneling effect becomes feasible at thicker films, approximately 10 nm,^52^ as opposed to films in the range of 1–5 nm. It is important to note that we already have weak crystallite TiO_2_ films coated via the spray method.^53−55^ Therefore, depositing sputtered thicker films above 5 nm may hinder the tunneling effect. Overall, the amorphous nature of TiO_2_-sputtered film (see Figure S1) reinforce the passivation layer effect of spray-coated TiO_2_. It is worth noting that most metal oxide photoanodes are typically based on crystalline phases.^56^ On the other hand, the most effective passivation layers are amorphous in nature due to their lack of crystal anisotropies and the absence of defects such as grain boundaries.^57,58^ Furthermore, amorphous coatings are more commonly used due to their excellent uniformity and conformality.^59−61^ Therefore, the TiO_2_ processed at sputtering technique retains amorphous nature, which serves as a protective layer, preventing the oxidation of the catalyst surface during PEC reactions. Theoretical studies conducted by Choi et al.^62^ reveal that crystalline–amorphous (c–a) junctions function as charge-separating heterojunction systems, thereby enhancing the PEC reactivity of semiconductors. In particular, the texturing of the c–a boundary plays a pivotal role in extending the lifetime of photocharge carriers. Consequently, the combined use of the integrated spray (with weak crystallinity) and sputtered (amorphous) TiO_2_ passivation layers provides an effective balance between charge separation and passivation effects.

We conducted performance tests on two configurations in our PEC experiments: (a) sputtered TiO_2_ alone on WO_3_/BiVO_4_ and (b) a sequential configuration with TiO_2_ sputtered as the first layer and spray-coated TiO_2_ as the top layer. The *J*–*V* results, as shown in Figure S5, indicate that sputtering TiO_2_ alone results in a lower PCD compared to having a spray-coated TiO_2_ passivation layer (refer to Figure 5a). Additionally, using sputtered TiO_2_ as the first layer exhibits lower photocurrent than the sequential coating order of spray-coated TiO_2_ followed by sputtered TiO_2_. This observation underscores the role of the spray-coated TiO_2_ layer as a seed layer^63^ for the sputtered TiO_2_ layer, enhancing the overall film integrity.

Simultaneously, we investigated the passivation effect of sputtered TiO_2_ on WO_3_/BiVO_4_, which exhibited a lower photocurrent compared to the photoanode with a spray-coated TiO_2_ passivation layer. This clearly underscores the integrated approach of combining a two-stage process involving spray and sputter-coated TiO_2_ films under optimized conditions. This approach provides conformal coverage on the BiVO_4_ surface to protect it from photocorrosion and allows photoholes to access the tunneling effect, thereby enhancing charge separation at the electrode/electrolyte interfaces. The charge separation effect in water splitting reactions has been further explored through incident photon-to-current efficiency (IPCE) analysis. Figure S4 presents the IPCE spectra of photoanodes containing WO_3_/BiVO_4_ and WO_3_/BiVO_4_/TiO_2_ (spray)/TiO_2_ (sputtering) layers. Notably, the photoanode with a TiO_2_ passivation layer demonstrates an impressive IPCE of approximately 70%, a significant improvement compared to the configuration without the passivation layer, which exhibits an IPCE of about 43%. This noteworthy enhancement in IPCE can be attributed to the charge separation occurring at both the bulk materials (WO_3_/BiVO_4_) and the interfaces between the photoanode and electrolyte. Researchers have extensively studied this phenomenon at the BiVO_4_ interface.^64−66^ The effectiveness of the TiO_2_ passivation layer in promoting charge separation is evident, showcasing its role in optimizing the PEC performance of the water splitting reaction.

**Figure 6:**
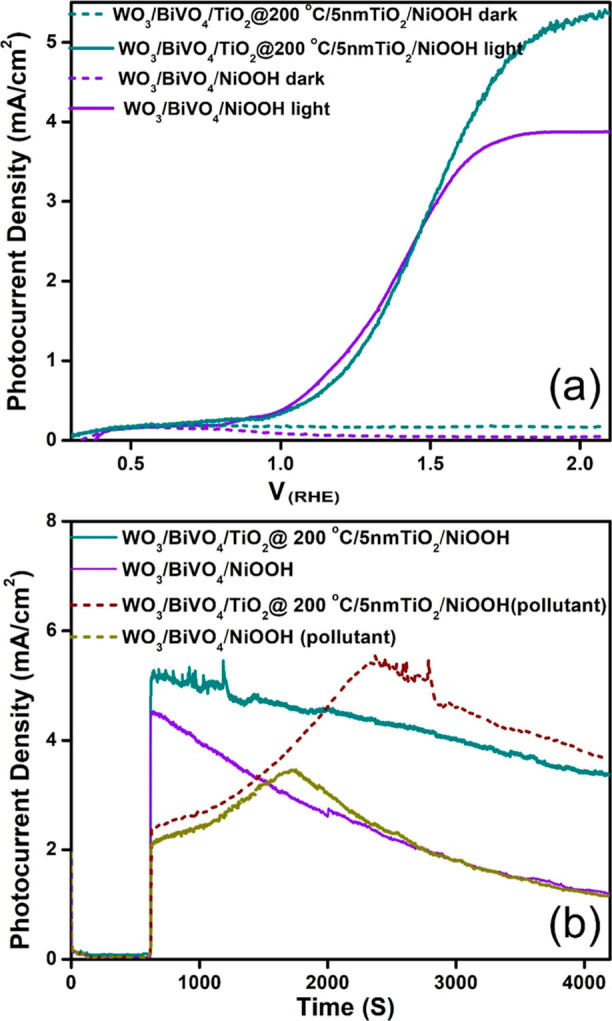
(a) *J*–*V* (current–voltage) plots of different photoanodes, demonstrating the influence of NiOOH cocatalysts on WO_3_/BiVO_4_. (b) Chronoamperometry of WO_3_/BiVO_4_/NiOOH and WO_3_/BiVO_4_/TiO_2_/5 nm TiO_2_/NiOOH photoanodes under various electrolyte conditions. The experiments were conducted with a two-compartment cells setup, where the photoanode compartment contained aqueous 0.5 M Na_2_SO_4_ electrolyte, and the cathode compartment utilized different electrolytes, including either aqueous 0.5 M Na_2_SO_4_ or real-time metal-mine-polluted water, without the addition of any inorganic salts.

Dark cocatalysts based on metal oxyhydroxides (M–OOH) hold promise in enhancing the photocurrent generation of photoanodes by mitigating charge accumulation at the surface of BiVO_4_, thereby preventing surface recombination effects. In a proof-of-concept study, we opted for NiOOH, a well-recognized champion cocatalyst for BiVO_4_, to further enhance the performance of our TiO_2_ passivation layer-coated photoanodes. The NiOOH layer was electrochemically deposited onto our optimized photoanode configuration: WO_3_/BiVO_4_/TiO_2_ (200 °C)/TiO_2_ (5 nm). We then assessed their photocurrent generation under both dark and light conditions (as depicted in Figure 6a). Notably, the photoanode coated with the NiOOH cocatalyst exhibits a PCD of approximately 5.3 mA/cm^2^ at 2 V RHE. This represents a 23% increase in PCD compared to photoanodes without the dark cocatalysts. However, an interesting question arises: what happens to the photocurrent generation when we apply cocatalysts to WO_3_/BiVO_4_ photoanodes without the TiO_2_ passivation layer? Figure 6a addresses this query, demonstrating that the NiOOH cocatalyst alone, when applied to the photoanode, results in a photocurrent of 3.8 mA/cm^2^, which is 39% lower than that of the TiO_2_ passivation layer-coated photoanode. This underscores the significance of cocatalysts in minimizing surface recombination effects and enhancing water oxidation performance. Concurrently, the TiO_2_ passivation layer assumes a dual function by shielding the photoanode from photocorrosion and augmenting charge separation at the electrode/electrolyte interfaces. It is crucial to recognize, however, that employing NiOOH cocatalysts on a sputter-coated TiO_2_ passivation layer-based photoanode yields approximately 2.5 mA/cm^2^ (Figure S6), indicating a 47% reduction in current density compared to photoanodes with integrated spray and sputter-coated TiO_2_ passivation layers (Figure 6a). This suggests that the processing route for the passivation layer’s growth onto the BiVO_4_ layer plays a pivotal role in the PEC performance, overshadowing the impact of dark cocatalyst coating. For instance, the spray-coated TiO_2_ layer serves as a seed layer for the subsequent sputtered TiO_2_, ensuring effective coverage of the BiVO_4_ layer. This sequential process is pivotal for achieving a higher PCD in spray and sputter-coated TiO_2_ passivation layer photoanodes.

Several research studies have already explored the function of M–OOH cocatalysts in the context of WO_3_/BiVO_4_ photoanodes.^67−69^ In a recent study, Durrant and colleagues^70^ conducted an exclusive examination of the role of M–OOH–coated BiVO_4_ in PEC reactions under stationary conditions. Their findings revealed a significant phenomenon: the accumulation of holes at the surface of BiVO_4_ led to substantial losses due to the slower kinetics of water oxidation on BiVO_4_ compared to surface recombination. However, when M–OOH catalysts were applied to the surface of BiVO_4_, the transfer of holes from BiVO_4_ to the M–OOH layer was notably enhanced. This resulted in the spatial separation of the accumulated MOOH (+) species from the photogenerated electrons within BiVO_4_. Consequently, surface recombination in the BiVO_4_/Ni (Fe)OOH system was reduced compared to unmodified BiVO_4_.

Furthermore, when performing *J*–*V* measurements under chopping conditions (light on/off) (Figure S7), we observe that the passivation layer and cocatalyst deposition work synergistically to support the stability of the photoanode. Finally, the chronoamperometry results depicted in Figure 6b illustrate that the WO_3_/BiVO_4_/TiO_2_ (200 °C)/TiO_2_ (5 nm)/NiOOH photoanode maintains excellent stability during 1 h of operation, with minimal reduction in photocurrent. The minimal reduction in photocurrent could potentially be attributed to the bubbling effect observed in static PEC cells. In this scenario, the continuous generation of gases (hydrogen at the cathode and oxygen at the anode) may lead to the accumulation of bubbles, which could obstruct the catalytically active sites on the cathode surfaces and block the light absorption to the anode.^71^ This issue can be mitigated by conducting PEC reactions in flow cells, where appropriate pressure can be applied to facilitate the removal of bubbles. Moreover, the introduction of a gas diffusion layer in the cathode compartment may exacerbate the bubble effect, potentially leading to increased interference.^72,73^ Proper management of gas bubbles is paramount in ensuring the accuracy and reliability of the experimental setup. Consideration of strategies, such as optimizing the gas diffusion layer or adjusting the flow dynamics, is essential to mitigate the impact of bubbles on the overall performance of the PEC system.

Recently, we conducted investigations into the promising impact of mine wastewater specifically zinc (Zn), present in mine wastewater, on PEC reactions.^74^ The elevated conductivity of mine wastewater facilitates the transport of protons in the cathode compartment. In line with this work, we explored the practicality of utilizing real-time metal mine wastewater as an electrolyte feedstock. Remarkably, as shown in Figure 6b, our results indicate a higher level of photocurrent production when employing metal mine water-based electrolyte (at cathode compartment) as opposed to aqueous Na_2_SO_4_-based electrolyte. In our observations, during the initial 2400 s, photocurrent generation in the mine wastewater-based electrolyte exhibited a gradual increase, after which it reached saturation. This behavior contrasts slightly with that observed in the aqueous Na_2_SO_4_ electrolyte. The gradual increment in photocurrent generation in the mine wastewater-based electrolyte suggests the deposition of metal ions (Zn^2+^) onto the cathode surface analogy to the cathodic electrochemical deposition in metal recovery reactions.^13,75^ These Zn^2+^ ions deposition may compete with catalytic sites for proton reduction, particularly in the generation of hydrogen gas. Once the cathode surface becomes saturated with metal ion deposition, the catalytic activity of the cathode tends to favor hydrogen generation.

We can further explain the rise in photocurrent in the metal mine wastewater-based electrolyte at the cathode is rooted in the substantially higher electrical conductivity, typically measured in 1–3 mS cm^–2^.^76^ This is primarily due to the presence of zinc ions (Zn^2+^). In comparison, the aqueous Na_2_SO_4_ electrolyte exhibits lower electrical conductivity, typically 10^3^ times lower than that of metal mine water. The disparity in electrical conductivity of electrolyte significantly influences the photocurrent generation. This phenomenon is analogy to conventional electrolysis for hydrogen generation, where a higher concentration of electrolyte supports effective ionic conductivity of hydroxyl ions (OH^–^) or hydrogen ions (H^+^), thereby facilitating a higher rate of water splitting reactions.^76,77^ Additionally, the present work involves PEC cells with different electrolytes at the anode (Na_2_SO_4_) and cathode (metal mine wastewater), resulting in distinct pH levels. The pH gradient created, with a lower pH at the cathode (pH 4–5) compared to the anode (pH 6), accelerates ion diffusion between anode and cathode. This gradient contributes to higher photocurrent generation in metal mine wastewater-based PEC cells.^78^ However, as discussed above, it is essential to note that this tendency persists only as long as heavy metals are present in the electrolyte. Once these metals are recovered onto the cathode surface, the conductivity of the mine wastewater becomes critical, leading to a subsequent reduction in its hydrogen generation performance.

**Figure 7:**
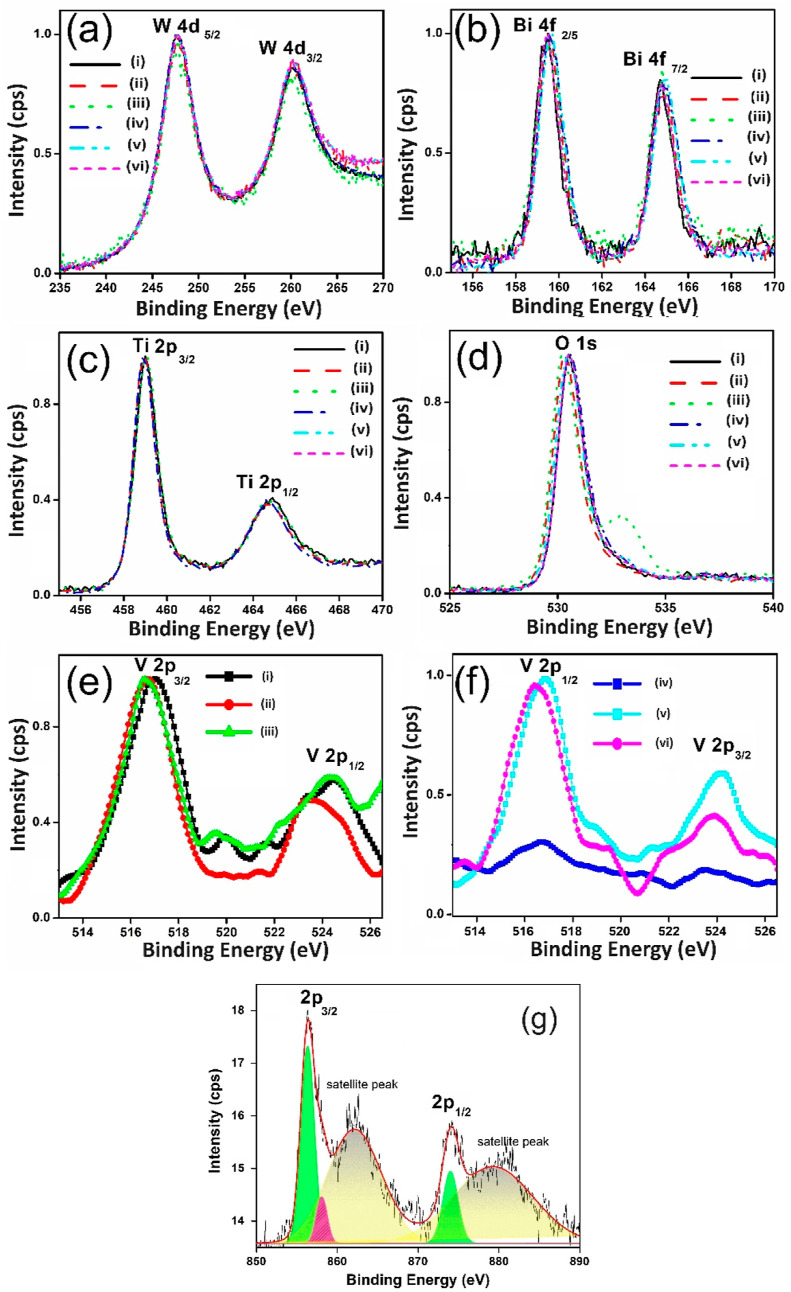
XPS spectra of (a) W 4d, (b) Bi 4f, (c) Ti 2p, (d) O 1s, (e) V 2p-before PEC experiments, (f) V 2p-after PEC experiments, (g) Ni 2p. The samples indicated with (i) WO_3_/BiVO_4_, (ii) WO_3_/BiVO_4_/TiO_2_@200 °C/5 nm TiO_2_, (iii) WO_3_/BiVO_4_/TiO_2_@200 °C/5 nm TiO_2_/NiOOH are fresh samples before PEC reactions. The samples mentioned with (iv) WO_3_/BiVO_4_, (v) WO_3_/BiVO_4_/TiO_2_@200 °C/5 nm TiO_2_, (vi) WO_3_/BiVO_4_/TiO_2_@200 °C/5 nm TiO_2_/NiOOH are the films after PEC reactions.

XPS spectra were conducted (Figure 7) to investigate the effectiveness of the TiO_2_ passivation layer and NiOOH cocatalysts coating in reducing the leaching of V^5+^ from the BiVO_4_ core during extended PEC reactions. To accomplish this, we examined six samples [referenced as (i–iv) in Figure 7] represents before and after PEC reactions for analysis. From Figure 7a–g, we observed major peaks corresponding to W 4d_5/2_ (247.6 eV), W 4d_3/2_ (260.3 eV) (Figure 7a), Bi 4f_5/2_ (164.7 eV), Bi 4f_7/2_ (159.4 eV) (Figure 7b), Ti 2p_1/2_ (464.7 eV), Ti 2p_3/2_ (458.9 eV) (Figure 7c), V 2p_1/2_ (524.4 eV), V 2p_3/2_ (516.8 eV) (Figure 7e), Ni 2p_1/2_ (874.1 eV), and Ni 2p_3/2_ (856.5 eV) (Figure 7f).^10,79−83^ These were attributed to oxidation states of +6, +3, +4, +5, and +3 for W, Bi, Ti, V, and Ni, respectively.^10,42−46^ The XPS spectra of O 1s in Figure 7d indicated an insignificant oxidation state of O (−2) with lattice oxygen (metal–O) at 530.5 eV, along with a shoulder at 532 eV confirming surface-adsorbed oxygen (–OOH). These results emphasized that the oxidation state of (a) W, (b) Bi, and (c) Ti remained relatively stable even after extended PEC reactions. However, the WO_3_/BiVO_4_ sample [referred to as (iv) in Figure 7f] exhibited a notable leaching of V^5+^ from the BiVO_4_ core during prolonged PEC reactions.

Conversely, in the NiOOH-coated WO_3_/BiVO_4_ film after PEC reaction, the shape of the V 2p core spectra was altered, indicating some degree of V^5+^ leaching from the BiVO_4_.^84^ In contrast, the spray and sputtered TiO_2_ passivation layer-coated BiVO_4_ samples, both before and after PEC reactions, exhibited unchanged V 2p spectra, suggesting the absence of V^5+^ leaching even after prolonged PEC reactions (Figure 7f). This stability contributes to the sustained water-splitting hydrogen generation observed in Figures 5d and 6b.

### 3.4 Solar to PEC Hydrogen Generation

**Figure 8:**
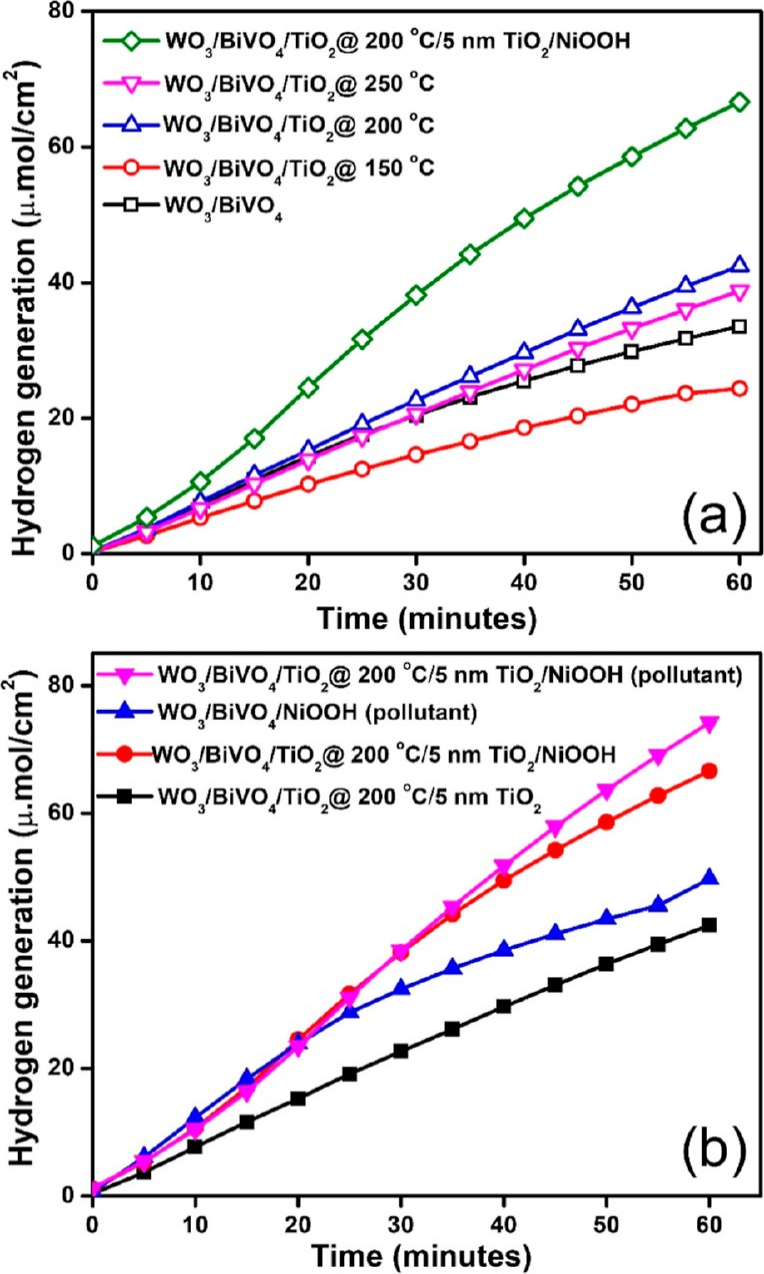
Quantification of H_2_ evolved during PEC reactions for different photoanodes and electrolyte feedstock. (a) H_2_ evolution from PEC experiments carried out using 0.5 M Na_2_SO_4_ (anode and cathode). (b) H_2_ evolution from PEC experiments carried out using 0.5 M Na_2_SO_4_ (anode) and mine polluted water at cathode. Note that the experiments were conducted with a two-compartment cells setup, where the photoanode compartment contained aqueous 0.5 M Na_2_SO_4_ electrolyte, and the cathode compartment utilized different electrolytes, including either aqueous 0.5 M Na_2_SO_4_ or real-time metal-mine-polluted water, without the addition of any inorganic salts.

We conducted measurements of hydrogen gas evolution through PEC reactions using different WO_3_/BiVO_4_ photoanodes equipped with passivation layers and cocatalyst depositions, and quantified the results with a gas chromatogram. The relationship between time and the quantity of hydrogen generated is illustrated in Figure 8. Notably, the champion configuration, WO_3_/BiVO_4_/TiO_2_@200 °C/TiO_2_ (5 nm)/NiOOH, yields a significantly higher amount of hydrogen gas, approximately 66.6 μmol/h cm^2^. Furthermore, this quantity experiences a remarkable enhancement when utilizing mine wastewater as a feedstock (74.2 μmol/h cm^2^). To the best of our knowledge, the overall hydrogen evolution rate (per hour per square centimeter) achieved in this study competes favorably with early reports on WO_3_/BiVO_4_ photoanodes.

Table 1 indicates that typically a WO_3_/BiVO_4_ photoanode demonstrates hydrogen gas evolution at a rate of approximately ∼20–60 μmol/h cm^2^, corresponding to a current density ranging from ∼2 to 6 mA/cm^2^. This rate can be further elevated to 70–80 μmol/h cm^2^ by introducing passivation layers or cocatalysts coatings. Moreover, the adoption of a tandem cell configuration serves to amplify the PCD, consequently enhancing the overall rate of hydrogen evolution.^92^

**Table 1:** WO_3_/BiVO_4_ Photoanode Performance in Water Splitting Hydrogen Generation with Various configurations

electrode	hydrogen generation μmol h^–1^ cm^–2^	reference
WO_3_/BiVO_4_	48.5	(39)
WO_3_/BiVO_4_	39.5	(85)
WO_3_/BiVO_4_/TiO_2_	40	(29)
Bi_2_S_3_/WO_3_	55	(86)
g-C_3_N_4_/BiVO_4_	2	(87)
Ag/AgCl/BiVO_4_	1.5	
metal–organic framework structure MIL-53(Fe) based on nanoporous 2% Mo/BiVO_4_	34.7	(88)
FeOOH/CQDs/CdIn_2_S_4_/BiVO_4_	38	(89)
Bi_2_MoO_6_/TiO_2_	21	(90)
g-C_3_N_4_/BiVO_4_	21.24	(91)
WO_3_/BiVO_4_/TiO_2_@200°C	42	this work
WO_3_/BiVO_4_/TiO_2_@200°C (spray)/NiOOH	66.4	this work
WO_3_/BiVO_4_/TiO_2_@200°C (spray)/5 nm TiO_2_ (sputtering)/NiOOH	74.2	this work

To assess the stability of the photoanode in PEC hydrogen generation reactions, we conducted a series of PEC experiments spanning four cycles. The hydrogen generation rate was measured per hour per square centimeter, and the outcomes are illustrated in Figure S8. The figure reveals a modest 5–10% reduction in hydrogen generation, attributed to the bubbling effect observed on the cathode surface. Despite this minor fluctuation, Figure S8 underscores the noteworthy stability achieved in PEC hydrogen generation. This stability serves as a testament to the robust performance of the benchmarked photoanode comprising WO_3_/BiVO_4_ with TiO_2_ passivation layers (spray and sputtered coating), complemented by NiOOH cocatalysts. These findings affirm the enduring performance and reliability of the photoanode in PEC applications.

**Figure 9:**
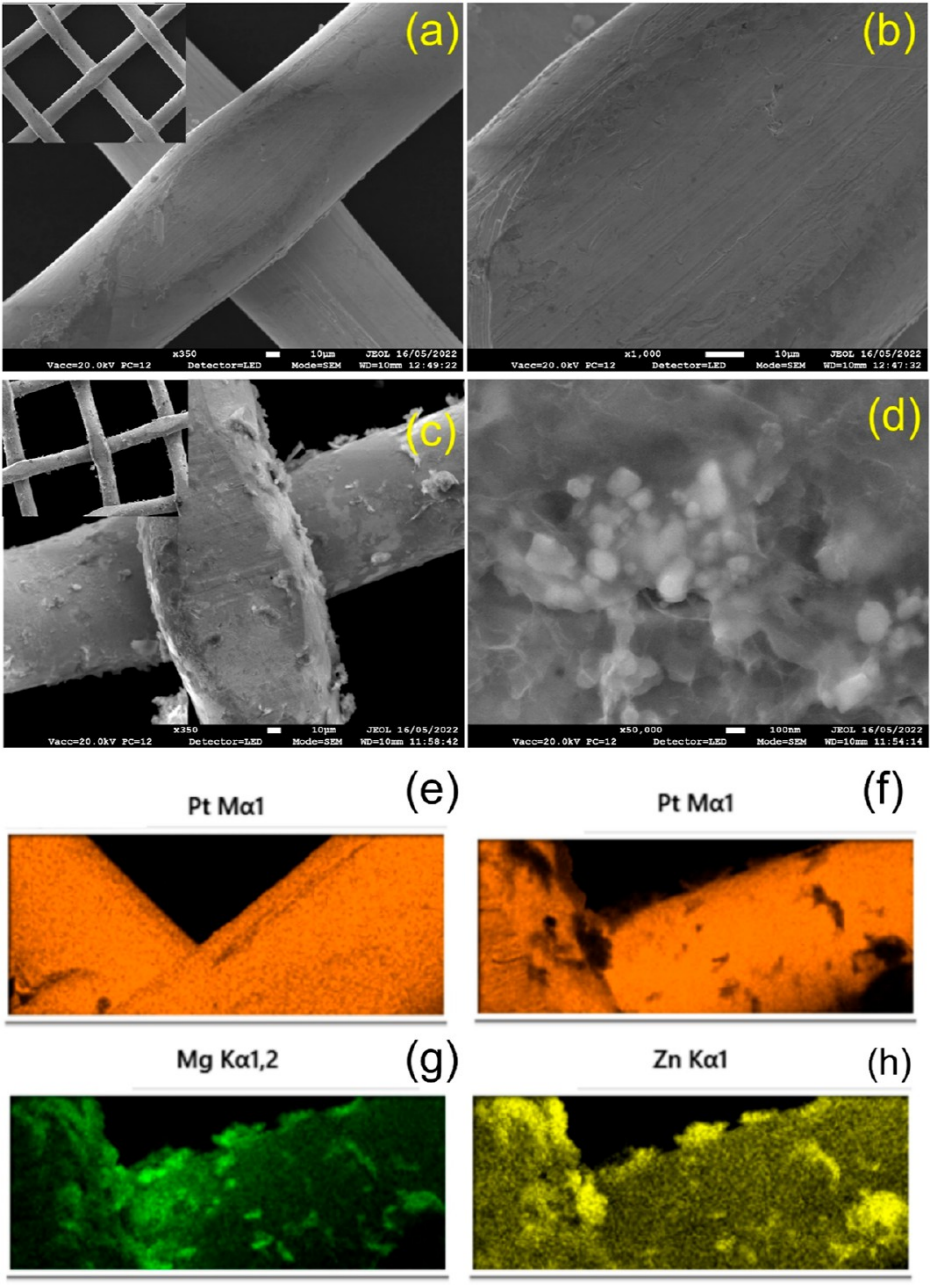
FESEM images of Pt mesh before the PEC reaction taken at different magnification scales (a) 10 μm, and (b) 10 nm. FESEM images of Pt mesh after the PEC reaction, captured at varying magnification levels (c) 10 μm, and (d) 100 nm. The insets of Figure 9a,c the FESEM images measured at 100 μm scale. The elemental mapping analysis of Pt mesh (e) before PEC reactions, and (f–h) represents after PEC reactions. Note that the PEC reaction involved with mine wastewater electrolyte at cathode compartment.

We verify the recovering Zn^2+^ metal ions through electrochemical deposition onto the cathode surface (Pt mesh) using SEM images, as depicted in Figure 9a–d. By comparing the SEM image of the pristine Pt mesh surface (Figure 9c) at a higher magnification of 100 nm to that of the Pt mesh surface after undergoing PEC processing (Figure 9d), we can clearly observe the deposition of Zn or ZnO particles. These particles were subjected to further analysis through EDS spectra, as shown in Figure 9e–h. The EDS spectra confirm the presence of ZnO coating on the Pt surface as a result of the PEC process, which is not present on the fresh Pt surface (refer to Figure S9).

**Figure 10:**
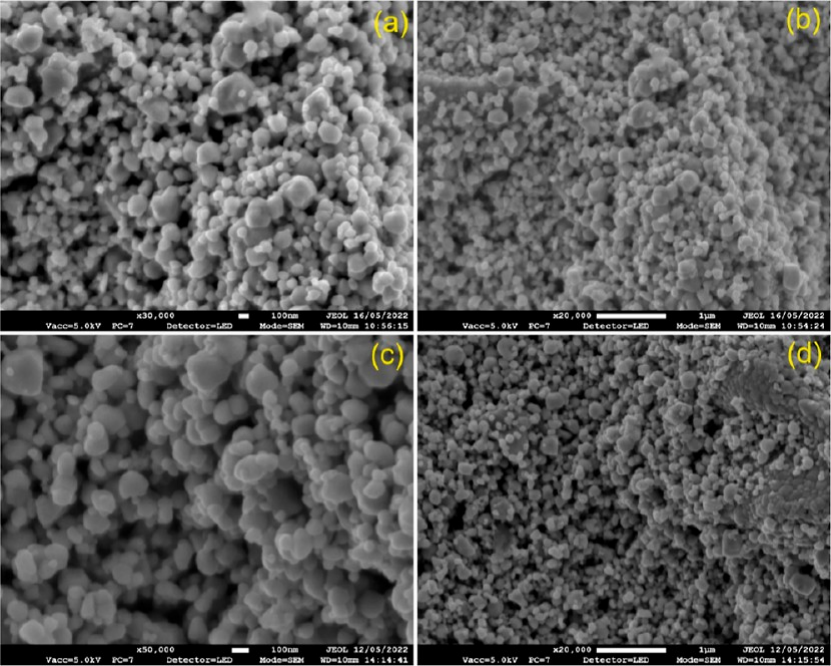
FESEM images of WO_3_/BiVO_4_/TiO_2_/5 nm TiO_2_/NiOOH photoanodes before PEC reaction measured at (a) 100 nm and (b) 1 μm. FESEM images of WO_3_/BiVO_4_/TiO_2_/5 nm TiO_2_/NiOOH photoanodes after PEC reaction measured at (c) 100 nm and (d) 1 μm.

To assess the photoanode’s stability in various electrolyte conditions, we conducted examinations both before and after PEC reactions, specifically following 1 h chronoamperometry studies. We utilized SEM images for this analysis (Figure 10a–d). Remarkably, the SEM images revealed that the WO_3_/BiVO_4_ particles remained unchanged after the PEC reactions, thanks to the protective influence of the TiO_2_ passivation layer and the presence of NiOOH cocatalyst deposition. This observation strongly suggests that WO_3_/BiVO_4_ photoanodes exhibit exceptional stability in water oxidation reactions.

**Figure 11:**
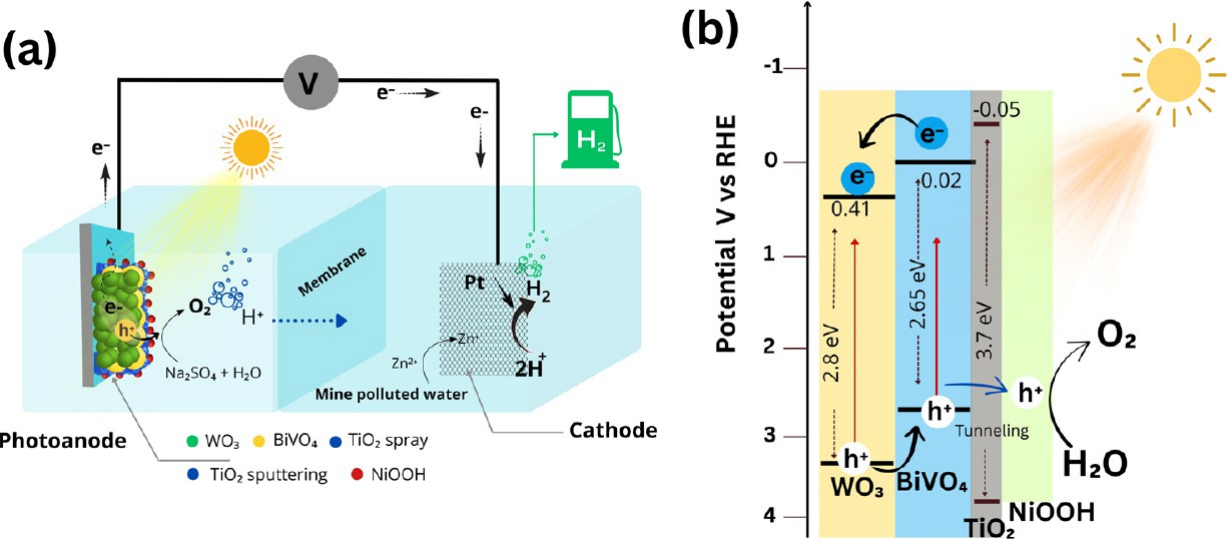
(a) Schematic illustration of PEC water oxidation and hydrogen generation using metal-mine-polluted water electrolyte (cathode), and WO_3_/BiVO_4_/TiO_2_/NiOOH photoanode, and Pt cathode. (b) Energetic structure of WO_3_/BiVO_4_/TiO_2_/NiOOH photoanode. The conduction band edge positions of WO_3_, BiVO_4_, and TiO_2_ has adopted from the others reports.^93,94^ Note that the band gap energy (eV) has estimated from diffused reflectance spectra (Figure S10a,b).

Based on the PEC results, Figure 11a illustrates the operating principle of PEC water splitting using WO_3_/BiVO_4_/TiO_2_ (spray)/TiO_2_ (sputter)/NiOOH. Upon light irradiation on the photoanode, photocharge carriers are generated. The photoholes are directed toward the electrolyte, initiating the oxidation of water to oxygen gas and protons (H^+^). These protons are then transported to the cathode via a proton exchange membrane. Simultaneously, the photoholes generated at the photoanode move to the cathode through a charge collector and circuit. These photoelectrons play a dual role by reducing protons into hydrogen gas and engaging in the reduction of Zn^2+^ ions present in mine water at the cathode, resulting in the deposition of Zn^+^ and facilitating metal recovery. In the photoanode, the combination of a spray and sputter-coated TiO_2_ passivation layer serves to protect against photocorrosion issues, ensuring sustainable operation. The efficiency of charge transfer at the photoanode and electrolyte interface is pivotal in determining the overall PEC performance of the cells. To provide a comprehensive understanding, Figure 11b offers a schematic illustration of WO_3_/BiVO_4_/TiO_2_ (spray)/TiO_2_ (sputter)/NiOOH.

In this illustration, photoelectrons excited from the valence band to the conduction band of BiVO_4_ are injected into the conduction band of WO_3_ before reaching the charge collector (FTO). The higher conduction band edge of BiVO_4_, compared to WO_3_, facilitates unidirectional electron transport flow from the point of photocharge carrier generation to the charge collector. Similarly, the photoholes at the valence band of WO_3_ are injected into BiVO_4_, eventually reaching NiOOH via tunneling transport through thin layers of TiO_2_. These photoholes catalyze the water oxidation reaction on the NiOOH surface. Notably, the higher conduction band of TiO_2_, compared to BiVO_4_, acts as a barrier, preventing electron transport from BiVO_4_ to the electrolyte. This enhances charge separation, thereby reducing the charge recombination rate at the photoanode/electrolyte interfaces.

## 4 Conclusions

In summary, we have successfully demonstrated the efficient and stable design of a WO_3_/BiVO_4_ photoanode through a comprehensive coating strategy involving doctor blade, spin-coating, spray, sputtering, and subsequent electrodeposition processes. This novel approach has resulted in enhanced PEC performance and hydrogen generation under simulated sunlight illumination. Our findings highlight the remarkable performance of the champion configuration, FTO/WO_3_/BiVO_4_/TiO_2_(200 °C)/TiO_2_(5 nm)/NiOOH multilayered photoanode, which exhibited a ∼88% enhancement in PCD of 5.38 mA cm^–2^ at 2 V compared to conventional FTO/WO_3_/BiVO_4_ (2.31 mA cm^–2^) at 2 V RHE. Post modification with a two-step TiO_2_ passivation layer and NiOOH catalyst led to approximately a 2-fold improvement in PEC water oxidation performance and, consequently, hydrogen generation-significantly surpassing the capabilities of WO_3_/BiVO_4_ alone. The combination strategy (sequential order) of a spray and sputter-coated TiO_2_ overlayer with optimized conditions played a crucial role in film growth, blocking surface defects and enhancing the surface charge carrier separation efficiency during PEC water splitting processes. This represents a pioneering achievement in the field of WO_3_/BiVO_4_ photoanodes.

Furthermore, we investigated the impact of NiOOH dark cocatalyst deposition on the PEC performance of WO_3_/BiVO_4_ photoanodes, comparing scenarios with and without passivation layers. This study underscores the critical role of the passivation layer in ensuring photoanode stability in the presence of NiOOH cocatalysts. Additionally, we explored the feasibility of utilizing real-time mine wastewater as a feedstock, which demonstrated the capability to produce hydrogen gas (74.2 μmol/h cm^2^) and recover metals (Zn^2+^ and Mg^2+^). In conclusion, our work sheds light on the innovative design of photoanodes, involving the fabrication of multilayered semiconductors with strategically ordered passivation layers and cocatalysts. This approach offers a pathway to achieving highly efficient and durable PEC water splitting for hydrogen generation.

## Supporting Information Available

The contents of SEM, EDS, *J*–*V* plots are supplied in the Supporting Information (PDF)

## Abbreviations

DRS: diffuse reflectance spectra
FTO: fluorine-doped tin oxide
GC: gas chromatography
PEC: photoelectrocatalysis
RHE: reversible hydrogen electrode
FESEM: field emission scanning electron microscopy
XPS: X-ray photoelectron spectroscopy
XRD: X-ray diffraction
PCD: photocurrent density
CVD: chemical vapor deposition
DC: direct current
RF: radio frequency
EDS: energy dispersion spectra
LSV: linear cyclic voltammetry
BID: barrier discharge ionization detector
UV–vis: ultraviolet–visible
