# Supplementary material for: Dual Shield: Bifurcated Coating Analysis of Multilayered WO3/BiVO4/TiO2/NiOOH Photoanodes for Sustainable Solar-to-Hydrogen Generation from Challenging Waters
Source: ACS Sustain Chem Eng. 2024 Feb 12;12(8):3044–60. doi: 10.1021/acssuschemeng.3c06528 (PMC10900524; doi:10.1021/acssuschemeng.3c06528)
Supplement: Supplementary file 1 — sc3c06528_si_001.pdf [file sc3c06528_si_001.pdf]

Supporting Information

Dual Shield: Bifurcated Coating Analysis of Multilayered $WO_3/BiVO_4/NiOOH/TiO_2$ Photoanodes for Sustainable Solar-to-Hydrogen Generation from Challenging Waters

Logu Thirumalaisamy,[a,b] Zhengfei Wei,[a] Katherine R. Davies,[a] Michael G. Allan,[c] James Mcgettrick, [a] Trystan Watson, [a] Moritz F. Kuehnel,[c,d] and Sudhagar Pitchaimuthu [a,e*]

a. SPECIFIC, Materials Research Centre, Faculty of Science and Engineering, Swansea University (Bay Campus), Swansea, SA1 8EN, UK.

b. Department of Physics, G T N Arts College, Dindigul, 624005, Tamil Nadu, India

c. Department of Chemistry, Swansea University, Singleton Park, Swansea, SA2 8PP, UK

d. Fraunhofer Institute for Microstructure of Materials and Systems IMWS, Walter-Hülse-Strasse 1, 06120 Halle, Germany

e. Research Centre for Carbon Solutions (RCCS), Institute of Mechanical, Processing and Energy Engineering, School of Engineering and Physical Sciences, Heriot-Watt University, Edinburgh, EH144AS, UK

S1. XRD analysis

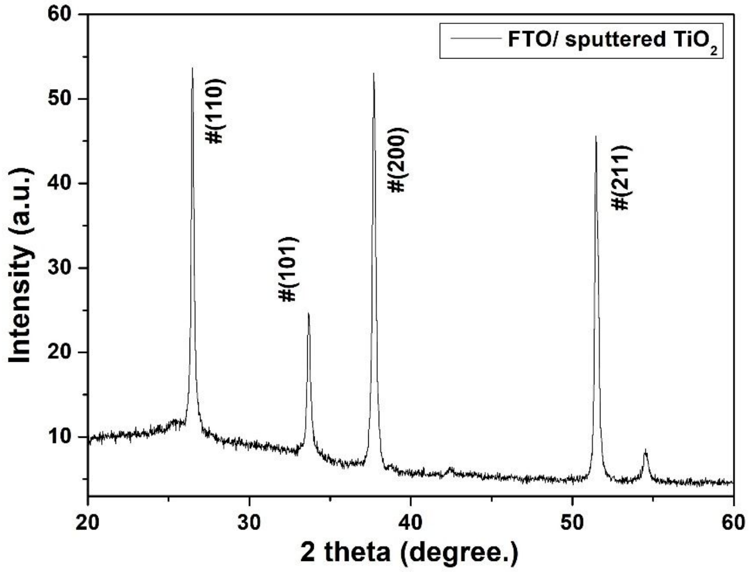

Figure S1. XRD results of 5nm TiO_2 sputtered thin films onto FTO substrate.

The crystallite peak observed in Figure S1 is associated with the FTO substrate. Notably, there is no evident peak corresponding to TiO_2 in the XRD analysis.

S2. Thin film thikcness measurements

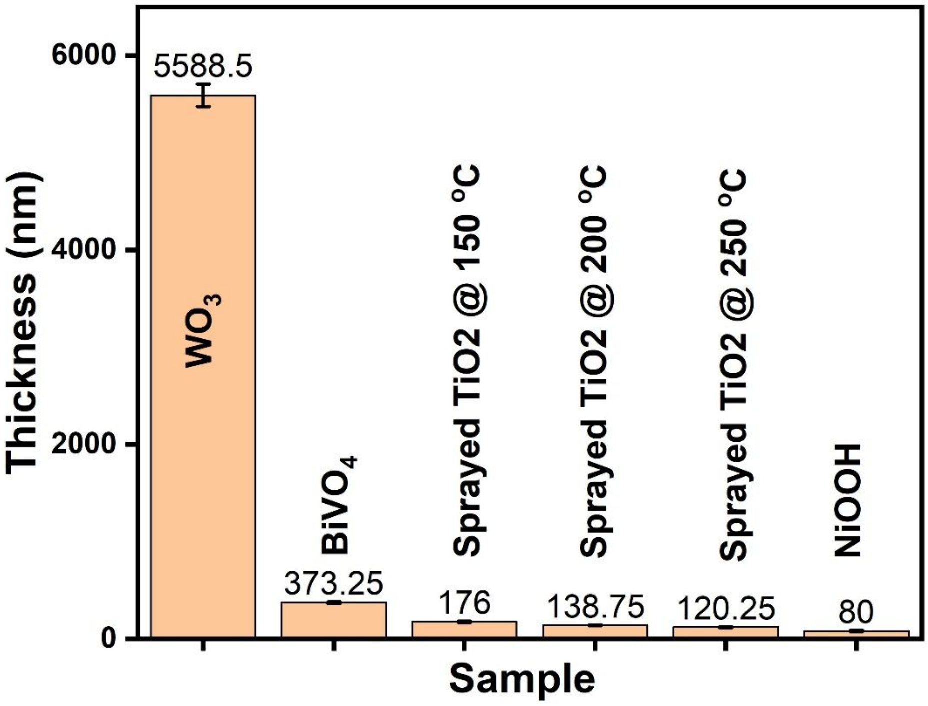

Figure S2. Surface profiler thickness data of the all individual layers used to construct the PEC Cell

The thickness of the coatings was measured using a stylus profilometer. The resulting values of the coatings are presented in Figure S2.

S3. Elemental analysis

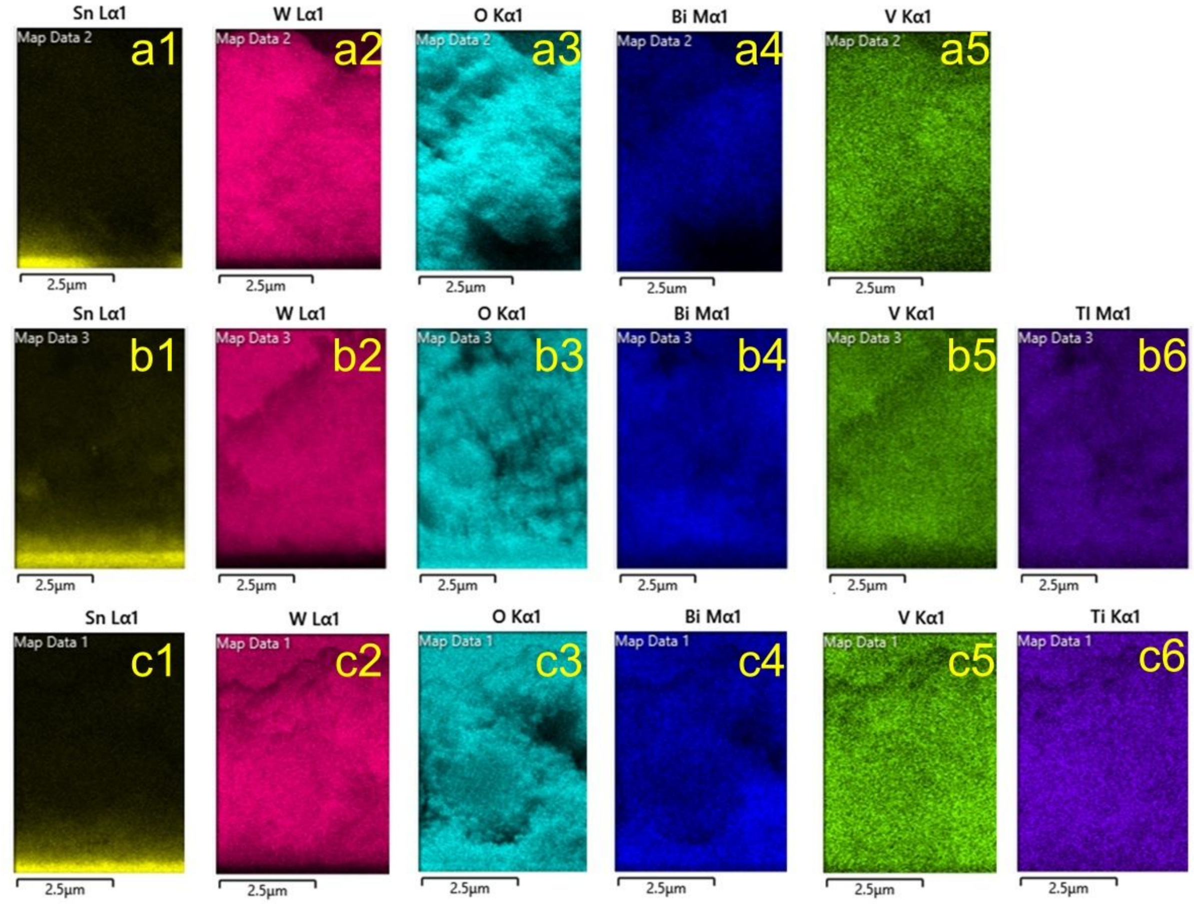

Figure S3. (a1-a5) SEM-EDX elemental mapping for Sn, W, O, Bi, V, and Ti elements on the $WO_3/BiVO_4$, (b1-b6) WO_3/ $BiVO_4/TiO_2$ @ 200 ºC and (c1-c6) WO_3/ $BiVO_4/TiO_2$ @ 250 ºC.

The elemental mapping of the various coatings was assessed using SEM-EDX. Figure S3 illustrates that the elements Bi, V, O, and W are uniformly distributed in the $WO_3/BiVO_4$ films prepared at different spray coating temperatures.

S4. Incident Photon-to-Current Efficiency (IPCE) analysis

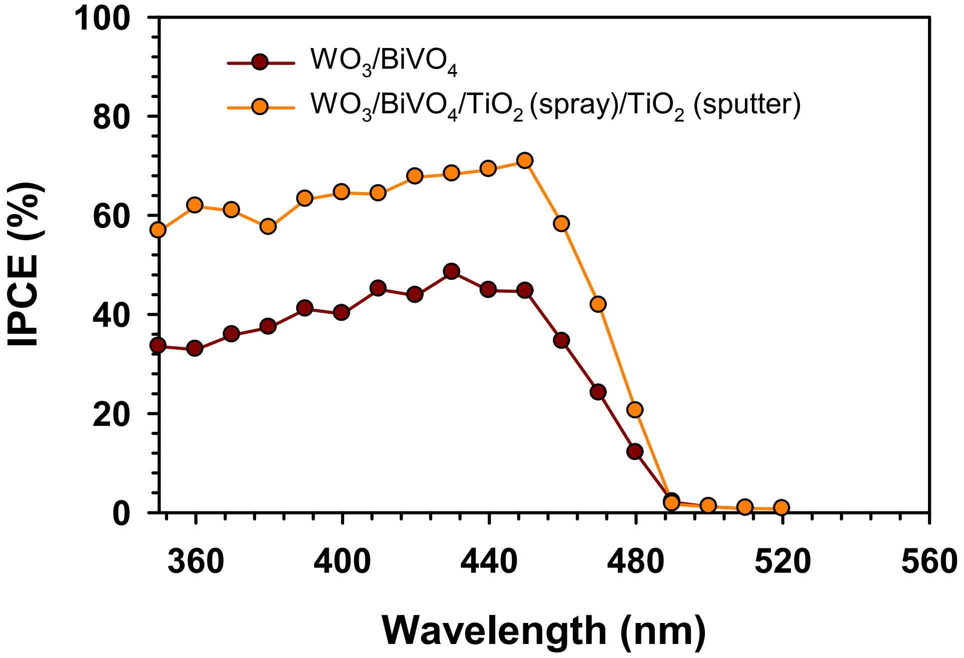

Figure S4: Incident Photon-to-Current Efficiency (IPCE) results depicting the impact of TiO_2 passivation layer coatings on $WO_3/BiVO_4$ photoanode performance, both with and without the passivation layer.

Figure S4 illustrates the Incident Photon-to-Current Efficiency (IPCE) results of $WO_3/BiVO_4$ films with and without TiO_2 passivation layer coatings. The figure demonstrates an enhanced IPCE (%) response of the $WO_3/BiVO_4$ photoanode with TiO_2 passivation layer coatings. This improvement may be attributed to enhanced charge separation, effectively addressing the photocorrosion issue.

S5. J-V experiments

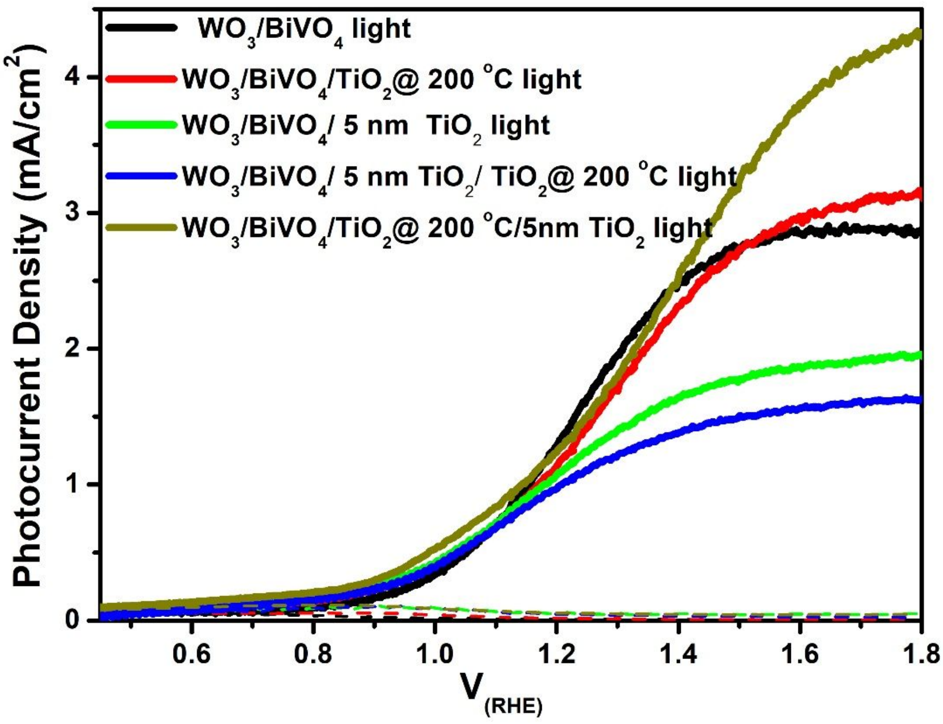

Figure S5. J-V resutls of TiO_2 sputtered films coated at different configuration photoanodes $WO_3/BiVO_4/5nmTiO_2$, $WO_3/BiVO_4$/5 nm TiO_2/ TiO_2@200 °C photoanodes. The results are compared with reference photoanode - $WO_3/BiVO_4$, benchmarking photoanode - $WO_3/BiVO_4/TiO_2$@200 °C/ 5 nm TiO_2.

Figure S5 illustrates the influence of passivation layers, specifically spray-coated TiO_2 (200°C) alone and a combination of spray-coated TiO_2 with sputter-coated TiO_2 (5nm), on overall photocurrent generation. The JV results presented in Figure S5 suggest that the combination of spray-coated TiO_2 (200°C) with sputtered TiO_2 yields a higher photocurrent density compared to pristine $WO_3/BiVO_4$ photoanodes and photoanodes coated with either one of the passivation layers.

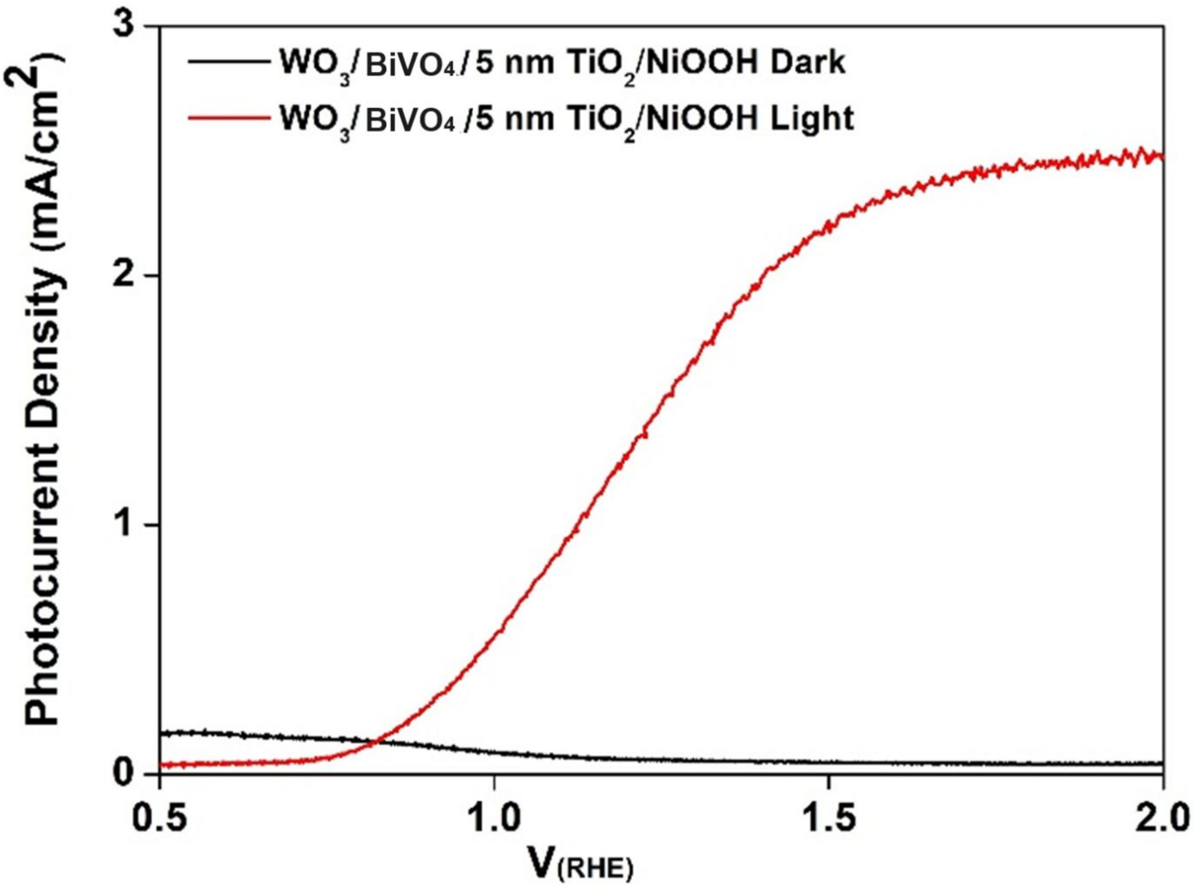

Figure S6. (a) J-V plot of PEC cells with $WO_3/BiVO_4/TiO_2$(sputter)/NiOOH. Note that the electrolyte is 0.5 M aqueous Na_2SO_4.

Figure S6 complements the findings presented in Figure S5. In Figure S6, NiOOH co-catalysts are coated on the $WO_3/BiVO_4$ photoanode without TiO_2 passivation layers. The results clearly indicate the crucial role of the TiO_2 passivation layer in enhancing photocurrent generation by addressing photocorrosion issues and facilitating efficient charge separation.

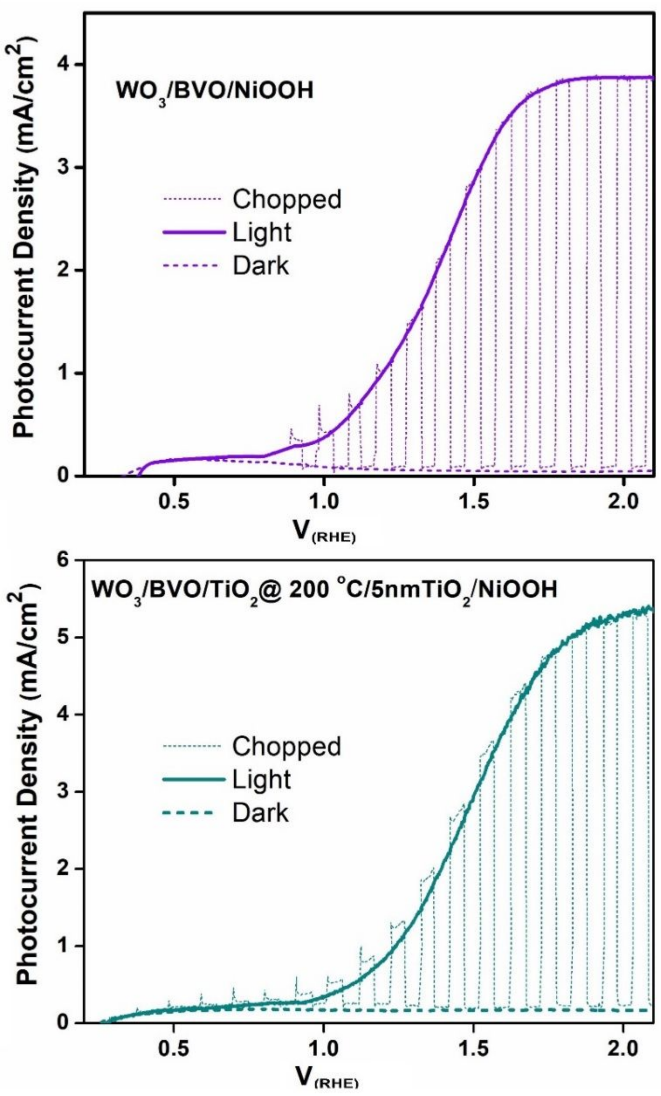

Figure S7. J-V resutls of $WO_3/BiVO_4/NiOOH$ and $WO_3/BiVO_4/TiO_2$@200 °C/5 nm TiO_2/ NiOOH photoanodes under chopping conditions.

The J-V measurements under chopping conditions (light on/off) are depicted in Figure S7. These results aid in comprehending the performance of the $WO_3/BiVO_4/NiOOH$ photoanode, benchmarked against the $WO_3/BiVO_4/TiO_2$@200 °C/5 nm TiO_2/ NiOOH photoanode, under alternating light and dark conditions. The consistent rise and fall in photocurrent observed in

both electrodes under light irradiation and dark conditions, respectively suggest effective control of charge recombination through the combined use of NiOOH and TiO_2 passivation layer coatings.

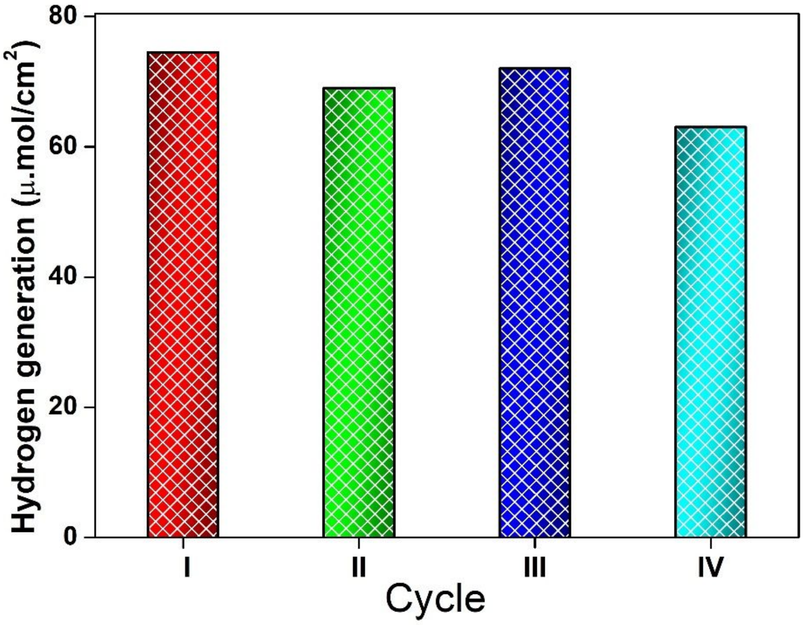

Figure S8. PEC hydrogen generation using $WO_3/BiVO_4$/Sprayed TiO_2@200 °C/5 nm TiO_2 (Sputtering)/ NiOOH photoanode for different cycles.

The sustainability of Photoelectrochemical (PEC) hydrogen evolution using the champion $WO_3/BiVO_4$/Sprayed TiO_2@200 °C/5 nm TiO_2 (Sputtering)/ NiOOH photoanode was investigated over four cycles. The outcomes of this study are illustrated in Figure S8.

S7. Elemental analysis of metal recovery

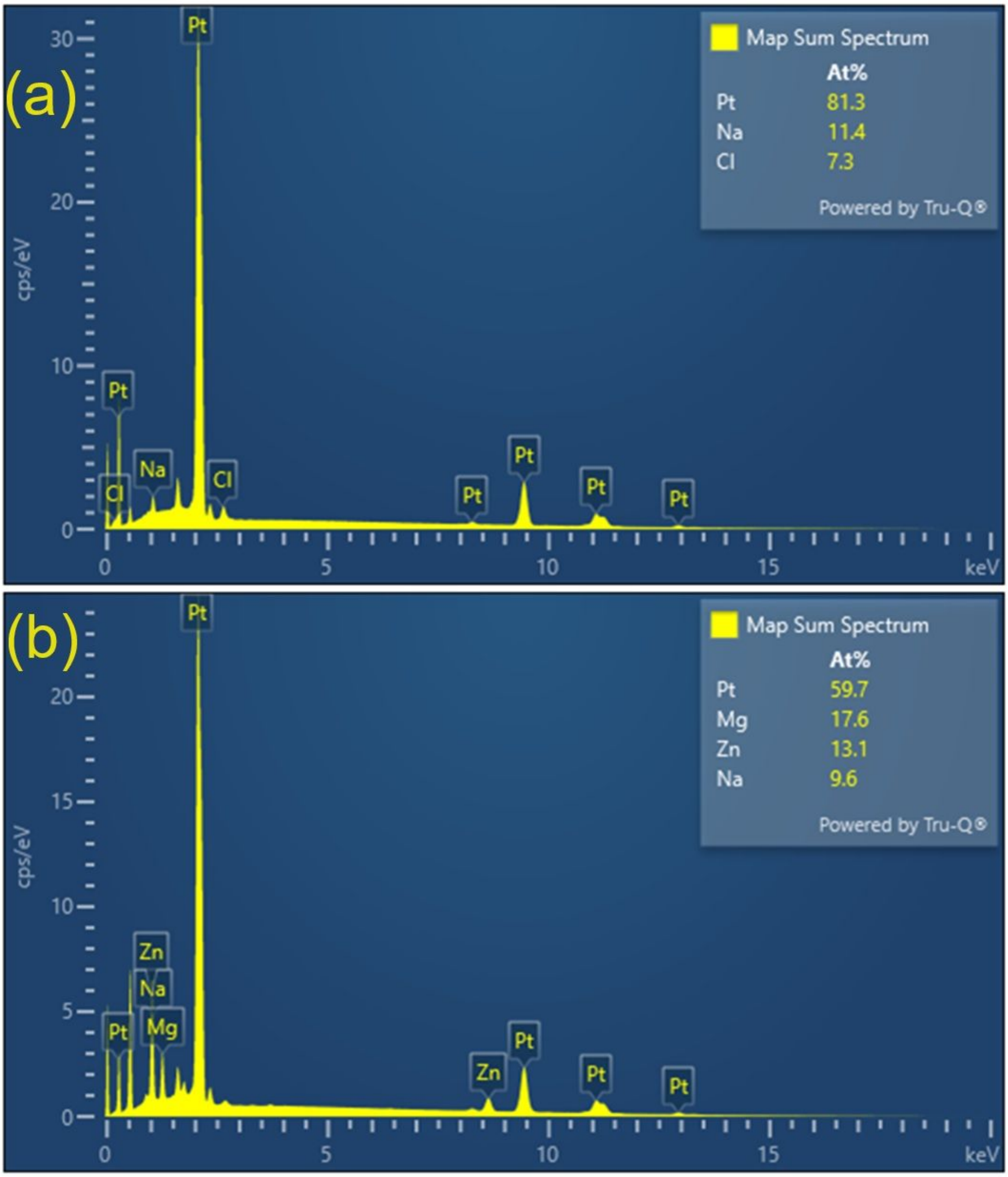

Figure S9. Elemental analysis of Pt mesh (a) before PEC reactions and (b) after PEC reactions.

Figure S9 elucidates the metal recovery (Zn^{+}) on the cathode surface before and after PEC reactions. Specifically, Figure S9(b) illustrates that zinc ions from the metal mine wastewater (catholyte) are coated on the cathode surface, as evidenced by the distinct Zn peaks.

S8. Bandgap estimation

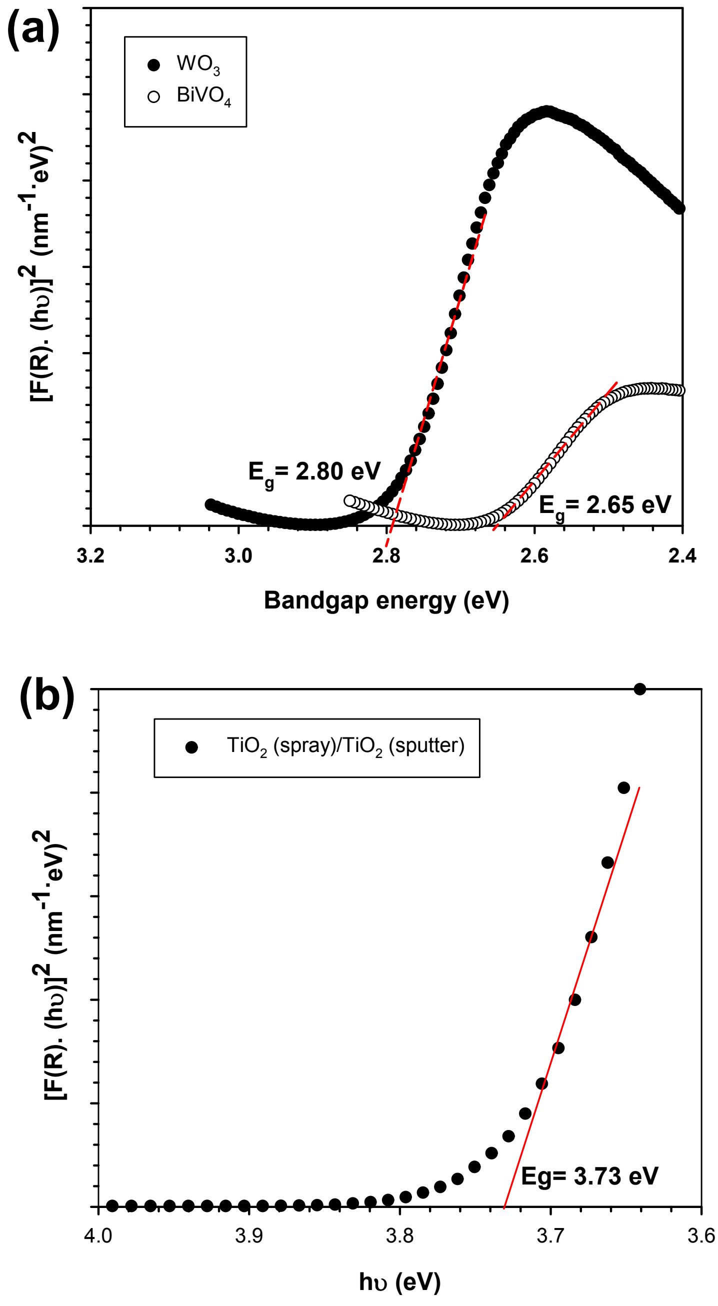

Figure S10. Bandgap analysis of (a) WO_3 and $BiVO_4$ films coated on FTO substrate and (b) TiO_2 films coated on FTO by spray (200 °C) and sputtering (5 nm) techniques.

The bandgap energy of individual coatings, namely WO_3, $BiVO_4$, and TiO_2, was estimated from the diffused reflectance spectra results and Tauc plots. The outcomes of these Tauc plots are presented in Figure S10.
